# The effect of a lifestyle intervention in obese pregnant women on gestational metabolic profiles: findings from the UK Pregnancies Better Eating and Activity Trial (UPBEAT) randomised controlled trial

**DOI:** 10.1186/s12916-018-1248-7

**Published:** 2019-01-21

**Authors:** Harriet L. Mills, Nashita Patel, Sara L. White, Dharmintra Pasupathy, Annette L. Briley, Diana L. Santos Ferreira, Paul T. Seed, Scott M. Nelson, Naveed Sattar, Kate Tilling, Lucilla Poston, Deborah A. Lawlor, On behalf of the UPBEAT Consortium

**Affiliations:** 1 MRC Integrative Epidemiology Unit at the University of Bristol, Oakfield House, Oakfield Grove, Bristol, BS8 2BN UK; 2 Population Health Science, Bristol Medical School, University of Bristol, Bristol, UK; 3 Division of Women’s Health, Faculty of Life Sciences and Medicine, King’s College London, London, UK; 4 School of Medicine, University of Glasgow, Glasgow, UK; 5 Institute of Cardiovascular and Medical Sciences, University of Glasgow, Glasgow, UK; 6 NIHR Bristol Biomedical Research Centre, Bristol, UK

**Keywords:** Pregnancy, obesity, metabolomics, lifestyle intervention, RCT

## Abstract

**Background:**

Pregnancy is associated with widespread change in metabolism, which may be more marked in obese women. Whether lifestyle interventions in obese pregnant women improve pregnancy metabolic profiles remains unknown. Our objectives were to determine the magnitude of change in metabolic measures during obese pregnancy, to indirectly compare these to similar profiles in a general pregnant population, and to determine the impact of a lifestyle intervention on change in metabolic measures in obese pregnant women.

**Methods:**

Data from a randomised controlled trial of 1158 obese (BMI ≥ 30 kg/m^2^) pregnant women recruited from six UK inner-city obstetric departments were used. Women were randomised to either the UPBEAT intervention, a tailored complex lifestyle intervention focused on improving diet and physical activity, or standard antenatal care (control group). UPBEAT has been shown to improve diet and physical activity during pregnancy and up to 6-months postnatally in obese women and to reduce offspring adiposity at 6-months; it did not affect risk of gestational diabetes (the primary outcome). Change in the concentrations of 158 metabolic measures (129 lipids, 9 glycerides and phospholipids, and 20 low-molecular weight metabolites), quantified three times during pregnancy, were compared using multilevel models. The role of chance was assessed with a false discovery rate of 5% adjusted *p* values.

**Results:**

All very low-density lipoprotein (VLDL) particles increased by 1.5–3 standard deviation units (SD) whereas intermediate density lipoprotein and specific (large, medium and small) LDL particles increased by 1–2 SD, between 16 and 36 weeks’ gestation. Triglycerides increased by 2–3 SD, with more modest changes in other metabolites. Indirect comparisons suggest that the magnitudes of change across pregnancy in these obese women were 2- to 3-fold larger than in unselected women (*n* = 4260 in cross-sectional and 583 in longitudinal analyses) from an independent, previously published, study. The intervention reduced the rate of increase in extremely large, very large, large and medium VLDL particles, particularly those containing triglycerides.

**Conclusion:**

There are marked changes in lipids and lipoproteins and more modest changes in other metabolites across pregnancy in obese women, with some evidence that this is more marked than in unselected pregnant women. The UPBEAT lifestyle intervention may contribute to a healthier metabolic profile in obese pregnant women, but our results require replication.

**Trial Registration:**

UPBEAT was registered with Current Controlled Trials, ISRCTN89971375, on July 23, 2008 (prior to recruitment).

**Electronic supplementary material:**

The online version of this article (10.1186/s12916-018-1248-7) contains supplementary material, which is available to authorized users.

## Background

Normal pregnancy is associated with marked changes in maternal metabolism, much of which is essential for healthy fetal growth and development, but may lead to adverse pregnancy, perinatal and longer term maternal and/or offspring outcomes if there is substantial deviation from physiological gestational levels [1–4]. It has been suggested that the metabolic changes seen in normal pregnancy differ in women who are obese and that these changes mediate at least some of the adverse short- and long-term outcomes associated with obese pregnancy [1, 5–8].

Randomised controlled trials (RCTs) in pregnant women have suggested some beneficial effect of lifestyle interventions on maternal gestational weight gain and adiposity [9, 10], but whether this improves their metabolic profiles is unclear. A recent RCT of a diet and physical activity lifestyle intervention in 376 obese (body mass index (BMI) ≥ 30 kg/m^2^) pregnant women found beneficial effects on gestational weight gain and C-reactive protein, but no evidence of benefit on insulin sensitivity/glucose tolerance or standard lipid (total cholesterol, very low-density lipoprotein (VLDL) cholesterol, low-density lipoprotein (LDL) cholesterol, high-density lipoprotein (HDL) cholesterol or triglyceride) measurements [11]. However, biomarkers were only assessed at two time points and analyses did not look at longitudinal change but treated results for each time-point as independent outcomes.

The UK Pregnancies Better Eating and Activity Trial (UPBEAT) RCT tested the effect of an intense behaviour change intervention in 1555 obese pregnant women (BMI ≥ 30kg/m^2^) on adverse pregnancy and perinatal outcomes [10]. The intervention was effective in reducing self-reported dietary intake of total energy, total fat, saturated fat and carbohydrates, as well as achieving a diet with a lower glycaemic load and index, and increasing protein and fibre intake as assessed at 28 weeks’ gestation [10]. Additionally, it led to a modest increase in the time spent walking and in the metabolic equivalent ratio of activity to rest, as well as reductions in gestational weight gain and adiposity, but it did not have an effect on the primary outcomes of gestational diabetes (GDM) or large for gestational age (LGA) neonates [10]. The addition of repeat (three occasions) gestational metabolic measurements in participants from this RCT provides a unique opportunity to determine the gestational metabolic profile in obese women and whether an intervention with known beneficial effects on diet, physical activity and adiposity influences this profile.

The aims of this study were to determine (1) how metabolic profiles change over gestation in obese pregnant women and (2) to assess the effects of an intervention that resulted in a healthier diet, increased physical activity, reduced gestational weight again and reduced adiposity in obese pregnant women, on change in gestational metabolic profiles.

## Methods

### Study design, randomisation and participants

UPBEAT was a multicentre RCT of a complex behavioural intervention of diet and physical activity advice versus standard antenatal care in obese pregnant women to prevent GDM and delivery of LGA neonates [10]. It involved eight inner-city centres in London (three centres), Bradford, Glasgow, Manchester, Newcastle and Sunderland. Approvals were obtained from the UK research ethics committee (UK integrated research application system, reference 09/H0802/5) and local Research and Development departments in participating centres approved participation of their respective centre; all women provided written informed consent prior to entering the study. UPBEAT is registered with Current Controlled Trials, ISRCTN89971375.

UPBEAT recruited and randomised 1555 obese women (BMI ≥ 30 kg/m^2^), aged 16 years or older and with a singleton pregnancy between 15^+0^ and 18^+6^ weeks of gestation (hereafter weeks). Exclusion criteria were multiple pregnancy, current use of metformin, unwilling or unable to provide written informed consent, or underlying medical disorders [10]. Women were randomised using an internet-based, computer-generated sequence that ensured concealment. In order to reduce differences between groups occurring by chance, the randomisation procedure included minimising by age, ethnic origin, centre, BMI and parity [10]. During pregnancy, women were followed up at 27^+0^ to 28^+6^ weeks (when an oral glucose tolerance test (OGTT) was completed) and at 34^+0^ to 36^+0^ weeks.

**Fig. 1:**
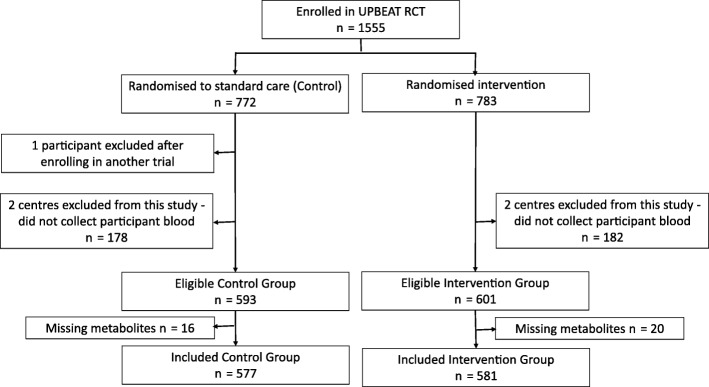
Participant flow

For the purposes of this study, women from two centres (King’s College Hospital, London, and Sunderland) were excluded as no blood samples were taken from participants in these centres for resource and logistical reasons (*n* = 360 in total; 178 and 182, respectively, from control and intervention arm were excluded). From the remaining six centres, all women with at least one metabolic profile assessment were included. Of the 1194 from the remaining six centres, 1158 (97%) had at least one set of nuclear magnetic resonance (NMR) metabolic measurements together with complete data on all covariables and were included in our analyses; proportions included were similar for the intervention and control groups (Fig. 1).

### Metabolic profiling

Venous blood samples were taken on three occasions – at recruitment, prior to randomisation (median (IQR) gestational age, 17.0 (16.1–17.9) weeks), post-randomisation at the time of the OGTT (27.7 (27.3–28.1)), and during the third trimester (34.7 (34.3–35.1)). Samples taken prior to randomisation and in the third trimester were non-fasting; those taken as part of the OGTT were after an overnight fast. All blood samples were initially kept on dry ice, processed within 2-hours and then stored at – 80 °C until metabolic profiling was performed. Metabolic analyses were undertaken on serum in two batches (March 2015 and August 2015), with samples from the three timepoints randomly distributed across these batches. Sample processing was automated with a Gibson 215 liquid processor. The complete process is illustrated in Additional file 1: Figure S1. A total of 158 metabolic features were measured and quantified using an NMR targeted platform (http://www.computationalmedicine.fi/), including 129 lipid measures (lipoprotein particle subclasses, particle size, cholesterols, fatty acids and apolipoproteins), 9 glycerides and phospholipids, and 20 low-molecular weight metabolites including branched-chain and aromatic amino acids, glycolysis-related markers, and ketone bodies [12]. This platform has been used in several large-scale epidemiological studies [4, 13–16] and further details and a full list of the 158 metabolites are presented in Additional file 1: Figure S1, Box S1 and Table S1. All blood samples were processed by laboratory technicians blinded to participant data, including the allocated randomisation arm.

### Statistical analyses

Full details of the statistical modelling are provided in Additional file 1: Box S1. Multilevel (random intercept and random slope) models were used to analyse the repeatedly assessed metabolic traits [17, 18]. We restricted the timeframe of these models to 16–36 weeks so that we were not predicting beyond available data. These models provide an individual (predicted) level of each metabolite at 16 weeks (the intercept) and an individual slope, which we present as the change in each metabolite per 4-week increase in gestational age between 16 and 36 weeks. In all analyses, we controlled for the minimising variables used in randomisation (BMI, ethnicity, parity, age and clinic centre) [10]. An interaction term between time (gestational weeks) and randomised arm (control or intervention) was also included.

We focus our discussion and interpretation of all results on the magnitude of the point estimates (i.e. pregnancy change in metabolites or effect of the intervention) and their precision (i.e. 95% confidence intervals) as recommended by the American Statistics Society and others [19–21]. We explore the role of chance by providing *p* values after controlling for multiple testing using the false discovery rate using the method of Benjamini and Hochberg [22].

### Change in metabolic profiles across pregnancy in obese women in the control arm

We present the change in metabolic profiles between 16 and 36 weeks in standard deviation (SD) units in women who were randomised to the control arm of the trial. SD units were used to aid comparison of results between metabolites and with those from other studies. We also present the mean absolute differences in the original units of measure for each metabolite (mostly mmol/L) in the control arm. The full model results (mean intercept and mean slope per 4 weeks) for each metabolite in their original units are also presented for all 1158 women included in the analyses. As the model includes a term for the randomised arm for each woman, these can be interpreted as the mean level of each metabolite at 16 weeks, and its change per 4 weeks of gestation between 16 and 36 weeks having adjusted for any effect of the intervention. The slope is therefore an indication of mean rate of change in metabolites in obese women in general (i.e. without any intervention effect).

### Effect of the intervention on change in metabolic profiles across pregnancy

In the main analyses, we present the effect of the intervention on rate of change in metabolites in SD units per 4 weeks (using the SD value of the control group), for ease of interpretation and to enable the effects of the intervention to be compared across different metabolic measures. We also present the difference in mean rate of change in original units (mostly mmol/L per 4 weeks). We present exact *p* values for all results and focus our discussion of the effect of the intervention on point estimates and confidence intervals as recommended by the American Statistics Society [19]. We also indicate the role of chance by indicating, in figures, which results reached conventional *p* < 0.05 levels of statistical significance after controlling the false discovery rate using the method of Benjamini and Hochberg to deal with multiple testing [22].

### Analysis assumptions and sensitivity analyses

Repeat metabolite assessments occurred at three time-points within a narrow range of gestational ages, such that there are gaps of up to 10 weeks with no (or very little) data between each of the measurements (Additional file 1: Figure S2). Therefore, we had to use linear spline methods and could not explore smoothing methods or use fractional polynomials to determine the exact shape of metabolic trait change over pregnancy [18]. Furthermore, our main analyses assume that the effect of the intervention is consistent between the first two measurements (~16–28 weeks) and the second two (~28–36 weeks). To test this assumption, we modified the multilevel model to include the possibility that the magnitude of metabolite change might alter at 28 weeks, and visually compared the effect of the intervention for each trait between 16–28 and 28–36 weeks. The linear spline method we have used assumes the model residuals to be approximately normally distributed, which may not be the case. There is evidence that estimates of population average change, such as those we present here, are robust to non-normality in the residuals (for example, see [23]); however, these methods may not deal with skewness. Therefore, to explore this further, we have repeated our analyses of the effect of the intervention on differences in mean change of the metabolites using generalised estimating equations with robust standard errors and unstructured correlation matrices, which should be robust to non-normality and (some) misspecification of the working correlation matrices. The robust standard errors were calculated using the White–Huber sandwich estimator and are robust to heteroskedasticity*.* We undertook a set of 14 further sensitivity analyses using different approaches to examine the sensitivity of our conclusions to possible heteroskedasticity, skewness and data outliers. Additional details of the linear spline model and its assumptions, including assumptions related to missing repeat measurements, and these additional 14 sensitivity analyses are provided in Additional file 1: Box S1.

### Indirect comparison with metabolic profiles in unselected ‘healthy’ pregnancy

**Fig. 2:**
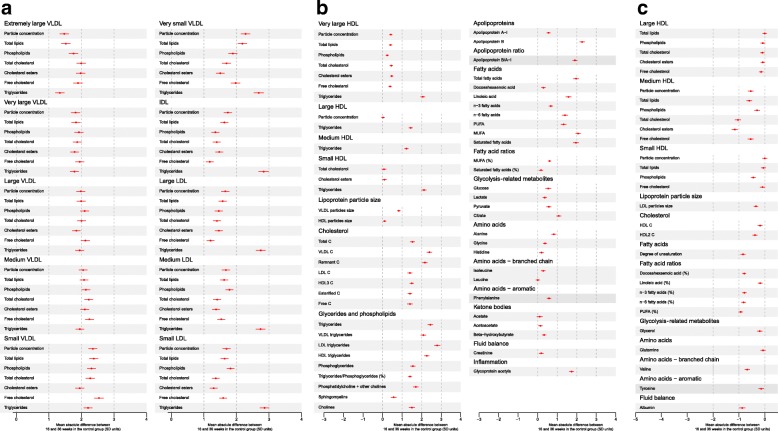
Mean change in metabolic measures between 16 and 36 weeks of gestation in obese pregnant women receiving standard antenatal care (*n* = 577). Mean increase in standard deviation units for all metabolites that (**a**, **b**) increase or (**c**) decrease across pregnancy. **a** Mean increase in all (extremely large, very large, large, medium, small and very small) VLDL particle concentrations; all IDL particle concentrations; all (large, medium and small) LDL particle concentrations. **b** Mean increase in some (those that increased across pregnancy; very large, large, medium and small) HDL particle concentrations; most cholesterols; all glycerides and phospholipids; both apolipoproteins; all glycolysis-related metabolites; those amino acids, ketone bodies, fluid balance and inflammatory markers that increased across pregnancy. **c** Mean decrease in most (large, medium and small) HDL particle concentrations; HDL cholesterol; degree of unsaturation of fatty acids, the percentage of total fatty acids that were docosahexaenoic acid, omega-3, omega-6, and polyunsaturated fatty acids; some amino acids; glycerol; and albumin. Circles are the mean change in SD units for each metabolic measure between 16 and 36 weeks’ gestation, with the horizontal lines representing the 95% CIs for this change. Numeric results of the absolute change between 16 and 36 weeks’ gestation for each trait in SD and original units for control women only are shown in Additional file 1: Table S3. The mean value of each trait at 16 weeks and rate of change per 4 weeks between 16 and 36 weeks’ gestation for all women (*n* = 1158), with adjustment for randomised group, are shown in Additional file 1: Table S4. *HDL* high-density lipoprotein, *IDL* intermediate-density lipoprotein, *LDL* low-density lipoprotein, *SD* standard deviation, *VLDL* very low-density lipoprotein

**Fig. 3:**
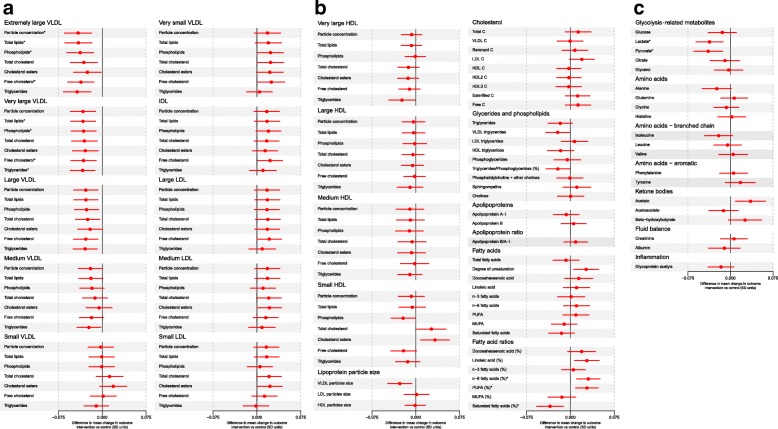
Effect of the UPBEAT intervention (difference in mean rate of change for each metabolic measure in SD units per 4 weeks of gestational age comparing those in the intervention arm to those receiving standard care) on mean rate of change in metabolic traits in SD units per 4 weeks (*n* = 1158). **a** The effect of the intervention for all (extremely large, very large, large, medium, small and very small) VLDL particle concentrations; all IDL particle concentrations; all (large, medium and small) LDL particle concentrations. **b** The effect of the intervention for all (very large, large, medium and small) HDL particle concentrations; lipoprotein particle sizes; all cholesterols; all glycerides and phospholipids; apolipoproteins; all fatty acids (absolute concentrations and percentages of total fatty acids). **c** The effect of the intervention for glycolysis-related metabolites; amino acids; ketone bodies; fluid balance and inflammatory markers. Circles are the difference in mean rate of change for each metabolic measure in SD units per 4 weeks of gestation comparing those randomised to intervention to those randomised to control (usual antenatal care) and horizontal lines are the 95% CIs for these differences. SDs were calculated from women in the control group. Results for the differences by randomised group for all traits in the original units (e.g. mmol/L) together with absolute *p* values are shown in Additional file 1: Table S5. * Results statistically significant at the conventional *p* < 0.05 after controlling for the false discovery rate. *HDL* high-density lipoprotein, *IDL* intermediate-density lipoprotein, *LDL* low-density lipoprotein, *SD* standard deviation, *VLDL* very low-density lipoprotein

We were keen to compare our findings in obese pregnant women to those in women not selected for being obese. As all of the participants in our study were selected for being obese, we were only able to do this indirectly by searching the literature for other studies of similar metabolite profiles in general populations of pregnant women. We identified one previous study, by Wang et al. [4], that examined cross-sectional differences using the same NMR metabolic profiles between women of reproductive age who were pregnant and those who were not (*n* = 4260 women; 322 of whom were pregnant). In addition to those cross-sectional analyses, longitudinal change in the metabolites was assessed in a subgroup of women (*n* = 583) who were either pregnant at baseline and not at a follow-up assessment 6 years later, or were not pregnant at baseline and were so 6 years later; the study also compared results separately by trimester of pregnancy. We compared the magnitude of longitudinal change and differences by trimester using summary data from Wang et al. (specifically the results shown in Figs. 1, 2, 3 and 4 of that paper) [4] with our results to obtain some insight into whether pregnancy-related metabolic change differed between obese and non-obese women.

## Results

Participant characteristics were similar between control and intervention arms as well as between the 97% eligible participants who had complete data and were included in our analyses and all eligible participants (Table 1); they were also similar when compared to all women who were randomised irrespective of whether or not they were from a centre where blood sampling was undertaken (Additional file 1: Table S2). All women with at least one metabolic profile measure were included in this study. In total, 62% of included women had all three measurements, 22% had two measurements and 16% had one. The proportion of those with a metabolic profile measure decreased over gestation in line with a small loss to follow-up in the main RCT, but this was similar in both the intervention and control groups (Table 1).

**Table 1:** Participant characteristics

	Participants with at least one metabolic profile analysed (analysis sample) *n* = 1158^a^		All eligible participants in the six contributing centres *n* = 1194^a^	
	Control *n* = 577	Intervention *n* = 581	Control *n* = 593	Intervention *n* = 601
BMI, *n* (%)				
30–34.9 kg/m^2^	273 (47.3)	287 (49.4)	279 (47)	296 (49.3)
35–39.9 kg/m^2^	203 (35.2)	177 (30.5)	209 (35.2)	185 (30.8)
≥ 40 kg/m^2^	101 (17.5)	117 (20.1)	105 (17.7)	120 (20)
Ethnicity, *n* (%)				
White	389 (67.4)	384 (66.1)	396 (66.8)	397 (66.1)
Asian	38 (6.6)	43 (7.4)	43 (7.3)	45 (7.5)
Black	120 (20.8)	127 (21.9)	123 (20.7)	130 (21.6)
Other	30 (5.2)	27 (4.6)	31 (5.2)	29 (4.8)
Parity, *n* (%)				
Primiparous	260 (45.1)	257 (44.2)	266 (44.9)	265 (44.1)
Multiparous	317 (54.9)	324 (55.8)	327 (55.1)	336 (55.9)
Age, *n* (%)				
< 25 years	97 (16.8)	85 (14.6)	100 (16.9)	90 (15)
25–29 years	141 (24.4)	165 (28.4)	147 (24.8)	169 (28.1)
30–34 years	187 (32.4)	174 (29.9)	188 (31.7)	182 (30.3)
≥ 35 years	152 (26.3)	157 (27)	158 (26.6)	160 (26.6)
Gestational diabetes, *n* (%)				
Yes	146 (25.3)	137 (23.6)	148 (25.0)	137 (22.8)
No	366 (63.4)	344 (59.2)	370 (62.4)	351 (58.4)
Missing	65 (11.3)	100 (17.2)	75 (12.6)	113 (18.8)
Centre, *n* (%)				
Bradford	19 (3.3)	22 (3.8)	25 (4.2)	28 (4.7)
Glasgow	130 (22.5)	132 (22.7)	131 (22.1)	134 (22.3)
Manchester	67 (11.6)	67 (11.5)	70 (11.8)	69 (11.5)
Newcastle	120 (20.8)	116 (20)	122 (20.6)	120 (20)
St George’s, London	53 (9.2)	55 (9.5)	54 (9.1)	57 (9.5)
St Thomas’s, London	188 (32.6)	189 (32.5)	191 (32.2)	193 (32.1)
First clinic visit				
*n* (%)	538 (93.2)	545 (93.8)	593 (100.0)	601 (100.0)
Median (IQR) gestation, weeks	17.0 (16.1–17.9)	17.0 (16.1–18.0)	17.0 (16.1–17.9)	17.0 (16.1–18.0)
Second clinic visit				
*n* (%)	500 (86.7)	477 (82.1)	591 (99.7)	598 (99.5)
Median (IQR) gestation, weeks	27.7 (27.3–28.1)	27.7 (27.3–28.1)	27.7 (27.3–28.3)	27.7 (27.3–28.1)
Third clinic visit				
*n* (%)	407 (70.5)	374 (64.4)	524 (88.4)	485 (80.7)
Median (IQR) gestation, weeks	34.7 (34.3–35.1)	34.6 (34.3–35.1)	34.7 (34.3–35.3)	34.7 (34.3–35.3)

^a^The 1158 participants whose results are in the first two columns are a subgroup of the 1194 whose results are presented in the last two columns. *n* number, *IQR* interquartile range

### Change in metabolic profiles in obese pregnant women

Absolute concentrations of all lipids, phospholipids and triglycerides in lipoprotein subclasses, with the exception of large, medium and small HDL, increased across pregnancy from 16 to 36 weeks in the obese women randomised to usual care (Additional file 1: Tables S3 and S4). Most of these changes were substantial, with lipids and phospholipids in all sizes of VLDL, intermediate density lipoprotein and LDL concentrations increasing by 1–3 SD, and concentrations of triglycerides in these lipoproteins increasing by 2–3 SD (Fig. 2a–c). Concentrations of very large HDL particles increased to between 0.3 and 0.5 SD, except for triglycerides, which increased by 2 SD. Concentrations of most large, medium and small HDL particles generally decreased by modest amounts (Fig. 2c), with the exception of triglycerides, which increased by 1.5–3 SD, and total and esterified cholesterol in small HDL, which increased by ~0.1 SD (Fig. 2a). Total, remnant, esterified and free cholesterol, total triglycerides, phosphoglycerides and cholines, and total, linoleic, omega-6, MUFA, PUFA and saturated fatty acids all increased by 1.5–3 SD between 16 and 36 weeks (Fig. 2b), with more mixed and modest changes in fatty acid ratios. VLDL particle size increased by 0.8 SD, HDL size by 0.1 SD and LDL particle size decreased by 0.3 SD. There were also increases in glucose (0.5 SD), lactate (0.4 SD), pyruvate (0.6 SD) and the inflammatory marker glycoprotein acetyls (1.7 SD) (Fig. 2b). Alanine, glycine, histadine, isoleucine, phenylalanine, creatinine and all of the ketone bodies increased (Fig. 2b), whereas glutamine, valine, tyrosine and albumin decreased (Fig. 2c).

Indirect comparisons suggest that the magnitudes of change across pregnancy in obese women are 2- to 3-fold larger than the difference in the same metabolic measures on becoming pregnant (from not being pregnant) in unselected healthy women assessed by Wang et al. [4].

### Effect of the lifestyle intervention on change in the metabolic profiles of obese pregnant women

The intervention resulted in reductions in the rate of increase of concentrations of all lipids, phospholipids and triglycerides in extremely large, very large, large and medium VLDL particles, except for total cholesterol and cholesterol esters in medium VLDL (Fig. 3a, Table 2, Additional file 1: Table S5). It also resulted in reductions in the rate of change of VLDL particle size, triglycerides in very large HDL, phospholipids in small HDL, and the ratio of total triglycerides to phosphoglycerides (Fig. 3b, Table 2, Additional file 1: Table S5). There were effects on fatty acids, with a lesser reduction in those receiving the intervention in the proportion of all fatty acids that were linoleic, omega-6 and polyunsaturated, so that levels of these beneficial fatty acids were higher in the intervention group, and reductions in the rate of increase in the proportion of saturated fatty acids (Fig. 3b, Table 2, Additional file 1: Table S5). Rates of increase in lactate, pyruvate and alanine were reduced, and of acetate increased, in those randomised to the intervention (Fig. 3c, Table 2, Additional file 1: Table S5).

**Table 2:** Effect of the UPBEAT diet and physical activity lifestyle intervention on selected^a^ metabolic traits; *n* = 1158

	Difference in mean rate of change in metabolic traits per 4 weeks of gestation between 16 and 36 weeks comparing women receiving intervention to control group (95% confidence intervals)^b^	
	In original units (see first column) per 4 weeks, median (IQR)	In SD units per 4 weeks,^c^ median (IQR)
Extremely large VLDL		
Concentration of chylomicrons and extremely large VLDL particles, mol/L	– 5.430 × 10^– 12^ (– 8.323 × 10^– 12^ to – 2.537 × 10^– 12^)	– 0.041 (– 0.065 to – 0.018)
Total lipids in chylomicrons and extremely large VLDL, mmol/L	– 0.001 (– 0.002 to – 5.322 × 10^– 4^)	– 0.041 (– 0.064 to – 0.018)
Phospholipids in chylomicrons and extremely large VLDL, mmol/L	– 1.457 × 10^– 4^ (– 2.287 × 10^– 4^ to – 6.274 × 10^– 5^)	– 0.038 (– 0.061 to – 0.015)
Total cholesterol in chylomicrons and extremely large VLDL, mmol/L	– 1.623 × 10^– 4^ (– 2.725 × 10^– 4^ to – 5.212 × 10^– 5^)	– 0.032 (– 0.055 to – 0.009)
Cholesterol esters in chylomicrons and extremely large VLDL, mmol/L	– 6.952 × 10^– 5^ (– 1.289 × 10^– 4^ to – 1.015 × 10^– 5^)	– 0.025 (– 0.049 to – 0.002)
Free cholesterol in chylomicrons and extremely large VLDL, mmol/L	– 9.276 × 10^– 5^ (– 1.468 × 10^– 4^ to – 3.868 × 10^– 5^)	– 0.037 (– 0.059 to – 0.014)
Triglycerides in chylomicrons and extremely large VLDL, mmol/L	– 8.450 × 10^– 4^ (– 0.001 to – 4.077 × 10^– 4^)	– 0.043 (– 0.066 to – 0.020)
Very large VLDL		
Concentration of very large VLDL particles, mol/L	– 2.919 × 10^– 11^ (– 4.656 × 10^– 11^ to – 1.183 × 10^– 11^)	– 0.033 (– 0.054 to – 0.012)
Total lipids in very large VLDL, mmol/L	– 0.003 (– 0.005 to – 0.001)	– 0.033 (– 0.054 to – 0.012)
Phospholipids in very large VLDL, mmol/L	– 4.618 × 10^– 4^ (– 7.453 × 10^– 4^ to – 1.782 × 10^– 4^)	– 0.032 (– 0.053 to – 0.011)
Total cholesterol in very large VLDL, mmol/L	– 4.869 × 10^– 4^ (– 8.058 × 10^– 4^ to – 1.681 × 10^– 4^)	– 0.031 (– 0.052 to – 0.009)
Cholesterol esters in very large VLDL, mmol/L	– 2.409 × 10^– 4^ (– 4.085 × 10^– 4^ to – 7.328 × 10^– 5^)	– 0.029 (– 0.051 to – 0.007)
Free cholesterol in very large VLDL, mmol/L	– 2.459 × 10^– 4^ (– 3.986 × 10^– 4^ to – 9.316 × 10^– 5^)	– 0.032 (– 0.054 to – 0.010)
Triglycerides in very large VLDL, mmol/L	– 0.002 (– 0.003 to – 7.781 × 10^– 4^)	– 0.034 (– 0.055 to – 0.012)
Large VLDL		
Concentration of large VLDL particles, mol/L	– 1.436 × 10^– 10^ (– 2.419 × 10^– 10^ to – 4.524 × 10^– 11^)	– 0.028 (– 0.049 to – 0.008)
Total lipids in large VLDL, mmol/L	– 0.008 (– 0.014 to – 0.003)	– 0.028 (– 0.049 to – 0.007)
Phospholipids in large VLDL, mmol/L	– 0.001 (– 0.003 to – 4.202 × 10^– 4^)	– 0.027 (– 0.048 to – 0.006)
Total cholesterol in large VLDL, mmol/L	– 0.002 (– 0.003 to – 3.658 × 10^– 4^)	– 0.025 (– 0.046 to – 0.004)
Cholesterol esters in large VLDL, mmol/L	– 6.322 × 10^– 4^ (– 0.001 to – 4.684 × 10^– 5^)	– 0.021 (– 0.042 to 5.042 × 10^– 4^)
Free cholesterol in large VLDL, mmol/L	– 9.788 × 10^– 4^ (– 0.002 to – 3.053 × 10^– 4^)	– 0.028 (– 0.049 to – 0.007)
Triglycerides in large VLDL, mmol/L	– 0.005 (– 0.009 to – 0.002)	– 0.029 (– 0.050 to – 0.009)
Medium VLDL		
Concentration of medium VLDL particles, mol/L	– 2.706 × 10^– 10^ (– 5.176 × 10^– 10^ to – 2.363 × 10^– 11^)	– 0.020 (– 0.041 to 4.479 × 10^– 4^)
Total lipids in medium VLDL, mmol/L	– 0.009 (– 0.017 to – 5.203 × 10^– 4^)	– 0.020 (– 0.040 to 0.001)
Phospholipids in medium VLDL, mmol/L	– 0.002 (– 0.003 to 2.571 × 10^– 5^)	– 0.018 (– 0.038 to 0.003)
Total cholesterol in medium VLDL, mmol/L	– 0.001 (– 0.004 to 6.072 × 10^– 4^)	– 0.012 (– 0.034 to 0.009)
Cholesterol esters in medium VLDL, mmol/L	– 3.934 × 10^– 4^ (– 0.001 to 6.747 × 10^– 4^)	– 0.006 (– 0.028 to 0.017)
Free cholesterol in medium VLDL, mmol/L	– 0.001 (– 0.002 to – 5.763 × 10^– 6^)	– 0.018 (– 0.039 to 0.002)
Triglycerides in medium VLDL, mmol/L	– 0.006 (– 0.010 to – 0.001)	– 0.023 (– 0.043 to – 0.002)
Other lipids, lipoproteins affected by the intervention		
Triglycerides in very large HDL, mmol/L	– 2.479 × 10^– 4^ (– 4.714 × 10^– 4^ to – 2.436 × 10^– 5^)	– 0.024 (– 0.046 to – 0.001)
Phospholipids in small HDL, mmol/L	– 0.002 (– 0.004 to – 1.363 × 10^– 4^)	– 0.022 (– 0.042 to – 0.001)
Mean diameter for VLDL particles, nm	– 0.031 (– 0.054 to – 0.008)	– 0.027 (– 0.048 to – 0.007)
Serum total triglycerides, mol/L	– 0.015 (– 0.031 to 8.645 × 10^– 4^)	– 0.017 (– 0.038 to 0.003)
Triglycerides in VLDL, mmol/L	– 0.015 (– 0.028 to – 0.002)	– 0.022 (– 0.042 to – 0.001)
Ratio of triglycerides to phosphoglycerides	– 0.005 (– 0.009 to – 6.198 × 10^– 4^)	– 0.022 (– 0.042 to – 0.001)
Estimated degree of unsaturation	0.001 (1.229 × 10^– 4^ to 0.002)	0.027 (0.006 to 0.048)
Ratio of 18:2 linoleic acid to total fatty acids, %	0.069 (0.013 to 0.126)	0.028 (0.007 to 0.049)
Ratio of omega-6 fatty acids to total fatty acids, %	0.077 (0.023 to 0.131)	0.031 (0.011 to 0.051)
Ratio of polyunsaturated fatty acids to total fatty acids, %	0.079 (0.020 to 0.138)	0.028 (0.009 to 0.047)
Ratio of saturated fatty acids to total fatty acids, %	– 0.049 (– 0.083 to – 0.015)	– 0.035 (– 0.058 to – 0.012)
Other traits affected by the intervention		
Lactate, mmol/L	– 0.017 (– 0.027 to – 0.006)	– 0.037 (– 0.061 to – 0.013)
Pyruvate, mmol/L	– 0.002 (– 0.003 to – 6.028 × 10^– 4^)	– 0.039 (– 0.064 to – 0.014)
Alanine, mmol/L	– 0.001 (– 0.002 to – 2.708 × 10^– 5^)	– 0.024 (– 0.050 to 0.002)
Acetate, mmol/L	3.081 × 10^– 4^ (7.541 × 10^– 5^ to 5.408 × 10^– 4^)	0.035 (0.009 to 0.062)

^a^Results are the difference in mean rate of change of each trait in original units (2^nd^ column) and SD units (third column) per 4-weeks of gestation between 16 and 36 weeks for selected metabolic traits where *p* < 0.05 after correction for multiple comparisons or because they are within the same broad group of metabolites and/or have similar magnitudes of associations (point estimates) as other metabolites with smaller *p* values. Complete results for all traits assessed in their original units, together with exact *p* values, are shown in Additional file 1: Table S5 and in SD units in Fig. 3 in the main paper

^b^95% CIs are exact and not adjusted for multiple testing

^c^SD units are based on the standard deviation values for each metabolite in the control group of women

*HDL* high-density lipoprotein, *IDL* intermediate-density lipoprotein, *IQR* interquartile range, *LDL* low-density lipoprotein, *n* number, *VLDL* very low-density lipoprotein, *SD* standard deviation

### Sensitivity analyses

Though statistical power is reduced in analyses comparing change between the first two and last two measurements, we found that the effect of the intervention appeared consistent on change between 16–28 and 28–39 weeks (Additional file 1: Figure S3). Results for the effect of the intervention on change in metabolites were the same in our main multilevel linear spline models and the generalised estimating equations with robust standard errors, suggesting that any non-normality or outliers have not notably affected our results (Additional file 1: Figure S4).

All additional sensitivity analyses led to broadly similar results to the main analyses, the generalised estimating equation method and each other, with correlations between estimates from all of the different methods being above 0.9 (Additional file 1: Table S6). There were differences in specific results that reached false discovery-corrected statistical ‘significance’, with some *p* values being slightly higher or lower in the sensitivity compared with our original methods. These differences in *p* values could be due to incorrect model distributional assumptions, influence of outliers, or because non-robust tests are more efficient for any outcomes that are normally distributed. Significance tests for mean differences in extremely large and very large VLDL concentrations between the intervention and control arm, in particular, were very similar across the different methods (a full set of results for all of these analyses can be found in Additional file 2, with some additional discussion in Additional file 1: Box S1).

## Discussion

We have demonstrated marked changes across pregnancy in lipid metabolic profiles, as well as in an inflammatory marker, in obese pregnant women. We also found modest changes across pregnancy in glucose, some amino acids, ketone bodies and metabolites, potentially reflecting fluid balance. Importantly, we have demonstrated that a lifestyle intervention that effectively improved diet and physical activity, and reduced gestational weight gain and adiposity in these women, resulted in improvements in most VLDL particles and VLDL size, in comparison to women randomised to usual care. There were also effects of the intervention on gestational changes in fatty acid profiles, with a reduction across pregnancy in the proportion of fatty acids that were linoleic, omega-6 and polyunsaturated, such that levels of these beneficial fatty acids were higher in the intervention group.

### Strengths and weaknesses of this study

Key strengths of our study are the application of an intention-to-treat analysis in a large, well conducted RCT, with concealed random allocation and blinded assessment of metabolic profiles. We have appropriately modelled repeat measurements. These analyses assume that any missing data on the metabolic profiles is missing at random (i.e. that the effects of the intervention in those with some missing metabolic profile data are the same as in those with complete data at all three time points). Given this is a well conducted RCT, and there was similar loss to follow-up and proportions with metabolic profiles at each assessment in the two arms of the trial, together with similar baseline characteristics between the two randomised arms, it is likely that this assumption is met. Whilst three repeat assessments of metabolites across gestation in a large RCT is unique, we were only able to fit linear spline multilevel models because metabolites were measured on just three occasions, with very little variation in gestational age at measurement. Therefore, fitting non-linear models, for example, using fractional polynomial or other ‘smoothing’ methods, is not possible [17, 18]. The consistency of findings between our main analyses, general estimating equations and eight additional sensitivity analyses using different methods addressing possible non-normality, heteroskedasticity, skewness and outliers, support the robustness of our modelling approach. Our main analyses also assume that changes in metabolites are linear across pregnancy between 16 and 36 weeks. We explored this by comparing change between 16 and 28 weeks to those between 28 and 36 weeks, with results being broadly consistent in these two time periods. However, we acknowledge that statistical power for these comparisons between these two time periods is limited and we cannot exclude non-linear effects across pregnancy. Replication of our findings in a similar sized or larger RCT is important to mitigate against these findings being due to chance. However, we are not aware of any other study with relevant data for this replication. The consistency of findings across similar lipoprotein subclasses provides some reassurance that findings are not solely due to chance.

### Strengths and weaknesses in relation to other studies

An earlier prospective cohort study of unselected (e.g. on the basis of BMI) women that used the same metabolic profiles (from the same NMR platform) as used here, found that lipids and lipoproteins differed by 1 SD on average between women who were pregnant and those who were not, with no difference in glucose levels between them [4]. In the subgroup of women with repeat measurements and in whom metabolic profiles were measured at least once whilst they were pregnant and at least once when they were not, the differences within these women (comparing when they were and were not pregnant) were consistent with the cross-sectional analyses comparing pregnant women to a separate group of women who were not pregnant. A comparison of our results to those of Wang et al. [4] indirectly suggests that the extent of change in lipids, glucose and inflammation across pregnancy in obese pregnant women is 2- to 3-fold greater than the change in an unselected group of healthy women on becoming pregnant. It also appears that the difference in lipids and lipoproteins between the third and second trimester observed by Wang et al. [4] was less marked (at most 1 SD) than seen herein (up to 3 SD). These comparisons support the hypothesis that metabolic profiles are more markedly disrupted in obese than non-obese pregnant women. However, some caution is required in assuming full support for this hypothesis, since Wang et al. [4] compared pregnancy to non-pregnancy and did not have repeat measurements during pregnancy within the same women. The full quantification of differences in metabolic profile changes across pregnancy between obese and non-obese pregnant women requires large prospective studies with repeat assessments of metabolic profiles in both obese and healthy weight women from the same underlying population; we are not aware of any such study currently taking place.

Previous RCTs of lifestyle advice or metformin in obese or overweight pregnant women have reported little or no effect on standard lipid measurements (total, LDL or HDL cholesterol, VLDL cholesterol or triglycerides) [24–26]. Differences in intervention intensity or type (with the previous lifestyle interventions being less intense than that used in UPBEAT), inclusion of overweight as well as obese women in some studies, and the limited number of lipids and other metabolites thus far explored make comparisons with our study difficult. Additionally, previous studies have used a mixture of fasting or non-fasting samples for metabolic measurements. Herein, the first and third trimester samples were non-fasting, in contrast to that from the second trimester. In previous analyses using the same NMR platform [14], we have shown high levels of consistency between associations of these metabolites with cardiovascular diseases using non-fasting and fasting samples. Whilst we acknowledge the limited power of these analyses, the similarity between the changes observed from the first to second trimester measures and those from the second to third trimester also suggests that whether the samples are fasting or not does not have a marked impact on the results.

### Implications of our findings

The impact of the UPBEAT intervention on VLDLs that we have shown here is potentially important for maternal and fetal/offspring health [1, 7]. The normal pregnancy increase in triglycerides and VLDL is thought to be more marked in women who are obese in pregnancy, as our findings suggest. This, in turn, may increase oxidative stress and maternal endothelial dysfunction, which has been implicated in the adverse influences of maternal obesity on pregnancy and perinatal outcomes [7]. That the intervention tested in this study reduces the increase in VLDLs and triglycerides in obese women might therefore have benefits on the risk of adverse pregnancy and perinatal outcomes.

Maternal gestational fatty acid status is essential for healthy fetal development as all essential fetal fatty acids must be obtained from mothers. Specifically, high circulating levels of long-chain polyunsaturated fatty acids are important for fetal development. Thus, the effect of the intervention in terms of increases in the proportion of polyunsaturated fatty acids and omega-6 fatty acids suggests that any impact on maternal lipids and fatty acids is unlikely to have an adverse impact on the developing fetus.

Whilst the UPBEAT intervention resulted in important beneficial changes in maternal diet, physical activity, gestational weight gain and adiposity, it did not affect the primary outcomes of GDM or LGA neonates [10]. Much more intensive changes are likely to be required to influence these outcomes, as suggested by the null findings from other lifestyle advice RCTs in obese or overweight pregnant women [11, 24, 25, 27], and two recent RCTs of the effect of metformin in obese pregnant women [26, 28]. Importantly, we have shown that the UPBEAT intervention, with its impact on beneficial lifestyle change, has potential beneficial effects on obese pregnant women’s lipid, lipoprotein and fatty acid change. Interestingly, the metabolic measures that the UPBEAT intervention benefits are those that differ between women with and without GDM [29]. Furthermore, in separate analyses [30], we have also shown that the dietary changes made as a result of the UPBEAT intervention persist postnatally to at least 6 months, and that the intervention results in lower levels of adiposity in offspring at 6 months, as assessed by skinfold thickness.

## Conclusions and potential impact

We have shown marked changes in the metabolic profiles of obese pregnant women, with beneficial effects on VLDL and fatty acid profiles following a lifestyle intervention that improved their diet and physical activity. In separate analyses [30], we have shown that dietary changes made by the mothers as a result of this intervention persist until at least 6 months and result in reduced adiposity in offspring at 6 months. With further follow-up of these participants we will be able to explore the extent to which the improvements in lipid profiles observed during pregnancy persist postnatally and result in lasting health benefits.

## Additional files

Additional file 1: **Box S1.** Description of methods for the NMR platform used to quantify metabolic profiles, and detailed description of all statistical methods, including assumptions of these. **Table S1.** Participant characteristics with additional detail compared to Table 1 in main paper. **Table S2.** Metabolic measures quantified by the NMR platform used for this study. **Table S3.** Absolute difference between 16 and 36 weeks of gestation for each metabolic trait in obese pregnant women who were randomised to the control arm of the UPBEAT RCT (*n* = 577). **Table S4.** Mean concentration at 16 weeks of gestation and mean rate of change in concentration per 4 weeks of gestational age between 16 and 36 weeks of gestation for each metabolic trait in obese pregnant women in the UPBEAT RCT (*n* = 115). **Table S5.** Effect of the UPBEAT diet and physical activity lifestyle intervention on metabolic profiles: difference in mean rate of change in metabolic traits (original units) between women receiving intervention and the control group (*n* = 1158). **Table S6.** Correlations between estimates of mean slope from different sensitivity analyses. **Figure S1.** Stages and methods used for NMR platform metabolic measures (adapted from Wurtz et al. [12]). **Figure S2.** Illustration of the timing of metabolite measurements. **Figure S3.** Comparison of the effect of the UPBEAT intervention between 16 and 28 weeks of gestation and between 28 and 36 weeks of gestation (*n* = 1158). **Figure S4.** Comparison of results from our main multilevel model analyses and sensitivity analyses using generalised estimating equations for the effect of the UPBEAT intervention on change in metabolites. (DOCX 2580 kb)

Additional file 2: Excel file of main analyses and sensitivity analyses results. (XLSX 132 kb)

## Abbreviations

BMI: body mass index
GDM: gestational diabetes
HDL: high-density lipoprotein
LDL: low-density lipoprotein
LGA: large for gestational age
NMR: nuclear magnetic resonance
OGTT: oral glucose tolerance test
RCT: randomised controlled trial
SD: standard deviation units
UPBEAT: UK Pregnancies Better Eating and Activity Trial
VLDL: very low-density lipoprotein
