# Supplementary material for: The effect of a lifestyle intervention in obese pregnant women on gestational metabolic profiles: findings from the UK Pregnancies Better Eating and Activity Trial (UPBEAT) randomised controlled trial
Source: BMC Med. 2019 Jan 21;17:15. doi: 10.1186/s12916-018-1248-7 (PMC6340185; doi:10.1186/s12916-018-1248-7)
Supplement: Supplementary file 1 — Box S1. Description of methods for the NMR platform used to quantify metabolic profiles, and detailed description of all statistical methods, including assumptions of these. Table S1. Participant characteristics with additional detail compared to Table 1 in main paper. Table S2. Metabolic measures quantified by the NMR platform used for this study. Table S3. Absolute difference between 16 and 36 weeks of gestation for each metabolic trait in obese pregnant women who were randomised to the control arm of the UPBEAT RCT (n = 577). Table S4. Mean concentration at 16 weeks of gestation and mean rate of change in concentration per 4 weeks of gestational age between 16 and 36 weeks of gestation for each metabolic trait in obese pregnant women in the UPBEAT RCT (n = 115). Table S5. Effect of the UPBEAT diet and physical activity lifestyle intervention on metabolic profiles: difference in mean rate of change in metabolic traits (original units) between women receiving intervention and the control group (n = 1158). Table S6. Correlations between estimates of mean slope from different sensitivity analyses. Figure S1. Stages and methods used for NMR platform metabolic measures (adapted from Wurtz et al. [12]). Figure S2. Illustration of the timing of metabolite measurements. Figure S3. Comparison of the effect of the UPBEAT intervention between 16 and 28 weeks of gestation and between 28 and 36 weeks of gestation (n = 1158). Figure S4. Comparison of results from our main multilevel model analyses and sensitivity analyses using generalised estimating equations for the effect of the UPBEAT intervention on change in metabolites. (DOCX 2580 kb) [file 12916_2018_1248_MOESM1_ESM.docx]

**SUPPLEMENTARY MATERIAL**

**The effect of a lifestyle intervention in obese pregnant women on change in gestational metabolic profiles: findings form the UK Pregnancies Better Eating and Activity Trial (UPBEAT) RCT.** Harriet Mills, Nashita Patel, Sara L White, Dharmintra Pasupathy, Annette Briley, Diana L Santos Ferreira, Paul Seed, Scott M. Nelson, Naveed Sattar, Kate Tilling, Lucilla Poston, Debbie A Lawlor, On behalf of the UPBEAT Consortium.

| **Content** | **Page(s)** | **Description** |
| --- | --- | --- |
| **Box S1** |  |  |
| NMR platform | 3 | Description of methods for the NMR platform used to quantify metabolic profiles |
| Statistical methods | 3-6 | Detailed description of statistical methods |
| **Tables** |  |  |
| Table S1 | 7-8 | Participant characteristics. This is an extension of Table 1 in the main paper showing characteristics in those in the control and intervention arm amongst all women who were randomised and included in the original RCT (N = 1554), the subgroup of these who were from the six centres originally randomised in which blood samples were collected (eligible sample for this study; N = 1194) and the sub-sample of those with a metabolic profile assessed on at least one occasion (analysis sample for this study; N = 1158). |
| Table S2 | 9-12 | List of NMR metabolomic measures assessed in this study, with their units of quantification |
| Table S3 | 13-20 | Absolute (total) difference in each metabolic trait between 16- and 36-weeks of gestation in women randomised to control (usual care) in original units (mostly mmol/l) and standard deviation (SD) units (using the SD of each metabolic trait from the 16-weeks measure). |
| Table S4 | 21-29 | Mean level of each metabolic trait at 16-weeks of gestation (intercept of multilevel model) and mean rate of change of each trait per 4-weeks between 16- and 36-weeks (slope of multilevel model) in women in control and intervention arms of the RCT, with adjustment for randomised arm. These results are presented in the original units (mostly mmol/l) in which traits were measures. |
| Table S5 | 30-36 | Difference in mean rate of change of each metabolic trait per week of gestation (16- to 36-weeks) between women allocated to intervention and those allocated to control (reference) groups. These are presented in the original units (per 4-weeks) in which traits were measured. These results quantify the effect of the intervention on change in metabolic traits across pregnancy in obese pregnant women. |
| Table S6 | 37 | Correlation coefficients for results from different sensitivity analyses |
| **Figures** |  |  |
| Figure S1 | 38 | Summary of NMR platform used in this paper |
| Figure S2 | 39 | Demonstration of the timing of metabolite assessment. This illustrates the marked time gaps between repeat measures meaning that it is not possible to fit smoothed/non-linear models |
| Figure S3 | 40-43 | Comparison of the effect of the intervention on mean rate of change in each metabolic trait between 16 and 28 weeks and that between 28 and 36 weeks (SD units per 4-weeks). |
| Figure S4 | 44 | Comparison of results multilevel model analyses and generalised estimating equations for the effect of the UPBEAT intervention on change in metabolites |
| **References** | 45 |  |

**Box S1**

**Nuclear Magnetic Resonance (NMR) Spectroscopy Methods**

The NMR spectroscopy methodology is summarised in **Figure S1** below and the metabolites that were quantified, together with the units in which they are quantified, are shown **Table S2**. This approach uses three molecular windows, (two that were applied to native serum and one to serum lipid extracts requiring minimal preparation) to quantify the 158 metabolic traits. The NMR-based metabolite quantification is achieved through measurements of three molecular windows from each serum sample.[[1](#_ENREF_1), [2](#_ENREF_2)] Two of the spectra (LIPO and LMWM windows) are acquired from native serum and one spectrum from serum lipid extracts (LIPID window). The NMR spectra are measured using Bruker AVANCE III spectrometer operating at 500 or 600 MHz. Measurements of native serum samples and serum lipid extracts are conducted at 37^o^C and 22^O^C, respectively.

The LIPO window represents a standard spectrum of human serum displaying broad overlapping resonances arising from lipid molecules in various lipoprotein particles. The LIPO data are recorded using 8 transients acquired using a NOESY-presat pulse sequence with mixing time of 10ms and water peak suppression. The LMWM window includes signals from various low-molecular-weight molecules. The LMWM spectrum is recorded using a relaxation-filtered pulse sequence that suppresses most of the broad macromolecule and lipid signals to enhance detection of small solutes. Specifically, a Carr-Purcell-Meiboom-Gill (CPMG) pulse sequence with a 78ms *T_2_*-filter and fixed echo delay of 403µs is applied using 24 transients. The LIPID window of the serum extracts is acquired with a standard 1D spectrum using 32 transients.

***QC and outputs***

The NMR spectra were analysed for absolute metabolite quantification (molar concentration) in an automated fashion.[[2](#_ENREF_2)] For each metabolite a ridge regression model was applied for quantification in order to overcome the problems of heavily overlapping spectral data. In the case of the lipoprotein lipid data, quantification models were calibrated using high performance liquid chromatography methods, and individually cross-validated against NMR-independent lipid data. Low-molecular-weight metabolites, as well as lipid extract measures, were quantified as mmol/l based on regression modelling calibrated against a set of manually fitted metabolite measures. The calibration data are quantified based on iterative line-shape fitting analysis using PERCH NMR software (PERCH Solutions Ltd., Kuopio, Finland). Absolute quantification cannot be directly established for the lipid extract measures due to experimental variation in the lipid extraction protocol. Therefore, serum extract metabolites are scaled via the total cholesterol as quantified from the native serum LIPO spectrum.

**Statistical Methods**

***Data preparation prior to modelling repeatedly assessed metabolic trait measurements***

Gestation week of clinic attendance was centred by subtracting 16-weeks from all values. 16-weeks was chosen as the nearest 4-week multiple to the average time of the first clinic (17.0-weeks). For some results absolute (total) change between 16- and 36-weeks are used; for others the rate of change per 4 weeks of gestation are used by dividing the time unit in the multilevel models by 4.

In order to ensure model convergence the data were scaled such that for each metabolite, if 25% were less than 0.5 every response was multiplied by a factor of 10 until this was not the case. Model results were then rescaled to their original units for presentation. The only metabolic trait for which this was not done was cholesterol esters in IDL, as this trait did not converge properly in the multilevel model when scaled in this way, but did converge when it was not scaled.

***Modelling repeat metabolic profile data***

Data were modelled using a multilevel model[[3](#_ENREF_3), [4](#_ENREF_4)] with fixed effects for BMI, ethnicity, parity, age group, clinic centre, time-point (centered on gestational age) and the interaction of randomised group (intervention vs control) and time-point, and with random intercepts and random slopes for the individual participants. We restricted the time frame to between 16 and 36 weeks of gestation so that we were not predicting beyond available data. Therefore, the intercept represents the concentration of a metabolic trait at 16-weeks and the slope the rate of change in the concentration of a trait per week of gestation between 16 and 36 weeks (divided by 4 in all analyses to give a rate of change per 4-weeks of gestation). In all analyses we controlled for the minimising variables used in randomization (BMI, ethnicity, parity, age and clinic centre).[[5](#_ENREF_5)] An interaction term between time (gestational weeks) and randomised arm (control or intervention) was also included.

We present exact p-values for all results but focus our discussion of the magnitudes of point estimates (i.e. pregnancy change in metabolites or effect of the intervention) and their precision (i.e. 95% confidence intervals) as recommended by the American Statistics Society and others.[[6-8](#_ENREF_6)] We explore the role of chance by providing exact p-values after controlling the false discovery rate using the method of Benjamini and Hochberg to deal with multiple testing.[[9](#_ENREF_9)]

***Metabolic profile change in obese pregnant women***

We estimated the absolute mean difference in each metabolite between 16- and 36-weeks by subtracting the predicted (from the multi-level model) value at 16-weeks from that at 36-weeks for each woman in the control group. These results are presented in standard deviation (SD) units, to aid comparison of results with those from other studies. In these analyses the magnitude of the SD for each metabolite was that from levels at 16-weeks. Thus, if the SD for one metabolite at 16 weeks is 0.5mmol/l, we divided levels at 36-weeks for that metabolite by 0.5mmol/l. We also present the mean absolute differences in the original units of measure for each metabolite (mostly mmol/l). These absolute difference results are presented in Table S3 below.

The full model results (mean intercept and mean slope per 4-weeks) for each metabolite in their original units are also presented for all 1158 women included in analyses. As the model includes a term for the randomised arm for each woman, these can be interpreted as the mean level of each metabolite at 16-weeks, and its change per 4-weeks of gestation between 16- and 36-weeks having adjusted for any effect of the intervention. The slope is therefore an indication of mean rate of change in metabolites in obese women in general (i.e. without any intervention effect). These results are presented in Table S4 below.

***Effect of a lifestyle intervention that changed diet and physical activity in obese pregnant women on change in their metabolic profiles***

In the multi-level model described above the interaction term between intervention group and gestation weeks represents the mean difference in the rate of change (slope) for each metabolic measure between 16- and 36-weeks between women in the intervention and control groups. It therefore provides the effect of the intervention on rate of change in metabolites. In the main analyses we express this in SD units per 4-weeks (using the SD value of the control group), for ease of interpretation and to enable effects of the intervention to be compared across different metabolic measures. We also present the difference in mean rate of change in original units (mostly mmol/l per 4-weeks). These results are presented in Table S5 below.

***Additional sensitivity analyses***

Our main analyses assume that change in metabolic trait concentrations across the three measurements are linear (i.e. the change between the first (16 weeks) and second (28 weeks) measurement is consistent with that between the second (28 weeks) and third (36 weeks) for each trait). To test this assumption we modified the multilevel model to include a knot point at the second clinic, giving two slopes (differences in metabolic trait concentration) for the periods time periods before and after the second clinic assessment – i.e. comparing the difference in rate of change for each metabolite between intervention and control group in SD per 4-weeks between 16- and 28-weeks to that between 28- and 36-weeks. The model had similar fixed and random effects to the main analyses. The two separate slopes over the two time periods of change (16- to 28-weeks and 28- to 36-weeks) came from two fixed effects time periods (instead of one) and two fixed effects interaction terms for treatment with these two time periods.

***Assumptions of the multilevel linear spline model***

Due to the nature of the data collection, we cannot explore the pattern of change between the three timepoints, as the data in the intervening periods is sparse (see Figure S2 below). Specifically, we had to use linear spline methods and could not explore smoothing methods, or use fractional polynomials [[3](#_ENREF_3), [4](#_ENREF_4)] to determine the exact shape of metabolic trait change over pregnancy. The linear spline method we have had to use assumes the model residuals are approximately Normally distributed, which may not be the case with our data. However, there is evidence that estimates of population average change, such as those we present here are robust to non-Normality in the residuals (for example see reference [[10](#_ENREF_10)]) and we have further explored this using generalised estimating equations with robust standard errors (as described in the main paper).

We conducted the following additional sensitivity analyses to explore how robust our results were to heteroskedasticity, skewed distributions and outliers:

1. Multilevel model using SD as the scale comparison. The parameter estimate should be unbiased but the standard errors may be affected by non-normality of the measures.
2. Multilevel model using IQR as the scale comparison. This analysis should be robust to non-normality in the original measure, and the parameter estimate should be unbiased but the standard errors may be affected by non-normality of the measures.
3. A paired t-test (final measure-first measure), using bootstrapping to obtain standard errors, and with the SD of the first measure (in the control group) used as the scale comparison. This analysis should be robust to non-normality in the differences (via bootstrapping). Furthermore, when removing outliers (see below) bootstrapping will ensure that the standard error is correct.[[11](#_ENREF_11)]
4. A paired t-test (final measure-first measure), using bootstrapping to obtain standard errors, and with the IQR used as the scale comparison rather than the SD. This analysis should be robust to non-normality in the original measure (using the IQR rather than the SD), and also robust to non-normality in the differences (via bootstrapping). Furthermore, when removing outliers (see below) bootstrapping will ensure that the standard error is correct.
5. A paired t-test (final measure-first measure), using bootstrapping to obtain standard errors, and with the Median Absolute Deviation (MAD) used as the scale comparison rather than the SD or IQR, as one reviewer[[11](#_ENREF_11)] suggested this was a more robust measure of deviation, though in our analyses the correlation between the MAD and SD was > 0.98 across all metabolites and the two gave virtually identical results.

Repeated 1-5 above but first removing all outliers (top and bottom) at each timepoint and by treatment group. We initially removed the top and bottom 1% in these analyses having considered the following: (i) it is important to only remove measures that are highly likely to be erroneous and not true values that are markedly different to the mean for the study population, as the latter may introduce selection bias; (ii) in this study we were exploring a binary exposure (randomisation to intervention or standard treatment) and would not expect a high proportion of erroneous results at the extremes to influence our findings; and (iii) when we used the MAD*3.5 threshold for defining outliers as suggested by Ramsey and Ramsey,[[12](#_ENREF_12)] for all of metabolites <1% were above this threshold (see final spreadsheet in Supplementary File 2). However, one of the reviewers[[11](#_ENREF_11)] preferred that we use the MAD-median rule applied to our data, which, gave a threshold of MAD*2.24. For the vast majority of metabolites this also resulted in fewer than 1% at top and bottom being removed*.*

Results across these 10-sesitivity analyses (the listed 5, with and without removal of the top and bottom MAD*2.24 defined outliers) were very similar with correlations between each other and with the main results all > 0.9. Removal of outliers did not notably alter any results.

Additionally, we undertook four further sensitivity analyse; median quantile regression and 75^th^ centile quantile regression, both with and without outliers removed based on the MAD*2.24 rule. The overall pattern of results were similar to those of the main analyses and all other sensitivity analyses, with some evidence of larger differences in the upper quartile of metabolites than around the middle 50% (see Supplementary File 2).

All results for these sensitivity analyses are shown in Supplementary File 2. **Table S6** below shows correlation coefficients between the main analyses and sensitivity analyses (these are all ≥ 0.9.

Multilevel models of change over time allow all participants with at least one measure to be included in analyses under the assumption that data are missing at random. The statistical term ‘missing at random (MAR)’ differs from missing completely at random (MCAR) in that it does not mean missingness is independent of all other characteristics. It means that conditional on the covariables included in the model (here age, parity, ethnicity, BMI and study centre) and the observed repeat measurements, the missing repeat measurements are not systematically different to those observed. This means that the effect of the intervention in those with some missing metabolic profile data is the same as in those with complete data at all three-time points conditional on the covariables included in the model. The MAR assumption would also be necessary if we restricted analyses to only those will all three repeat measurements (i.e. a complete case analysis). Whilst we cannot directly assess this assumption we feel that it is unlikely to be violated given most women had all three repeats, with just 16% having only one measure and that loss to follow-up in the trial was minimal and similar in both arms. The standard errors, and hence 95% confidence intervals, in these models take account of the greater random error of predicted levels at any time in those with just one or two of the repeat measurements**.**

***Statistical Code*** for all analyses presented in this paper can be found alongside a pre-peer review, pre-print version of the paper at http://biorxiv.org/content/early/2017/04/10/125740

**Supplementary Tables**

**Table S1: Participant characteristics (extended from Main Text Table 1)**

|  | **Participants with at least one metabolic profile analysed during pregnancy (analysis sample for this study).**  **N = 1158*** | | **All participants in the six centres with blood sampling (eligible sample for this study). N = 1194*** | | **Participants in all eight samples who were included in the original RCT (irrespective of whether blood samples were collected at their centre) N = 1554*** | |
| --- | --- | --- | --- | --- | --- | --- |
|  | **Control**  **N = 577** | **Intervention**  **N = 581** | **Control**  **N = 593** | **Intervention**  **N = 601** | **Control**  **N = 771** | **Intervention**  **N = 783** |
| **BMI (N (%))** | | | | | | |
| 30 to 34.9 kg/m^2^ | 273 (47.3) | 287 (49.4) | 279 (47%) | 296 (49.3%) | 374 (48.5) | 391 (49.9) |
| 35 to 39.9 kg/m^2^ | 203 (35.2) | 177 (30.5) | 209 (35.2%) | 185 (30.8%) | 262 (34.0) | 246 (31.4) |
| ≥40 kg/m^2^ | 101 (17.5) | 117 (20.1) | 105 (17.7%) | 120 (20%) | 135 (17.5) | 146 (18.6) |
| **Ethnicity (N (%))** | | | | | | |
| White | 389 (67.4) | 384 (66.1) | 396 (66.8%) | 397 (66.1%) | 483 (62.6) | 490 (62.6) |
| Asian | 38 (6.6) | 43 (7.4) | 43 (7.3%) | 45 (7.5%) | 48 (6.2) | 47 (6.0) |
| Black | 120 (20.8) | 127 (21.9) | 123 (20.7%) | 130 (21.6%) | 199 (25.8) | 202 (25.8) |
| Other | 30 (5.2) | 27 (4.6) | 31 (5.2%) | 29 (4.8%) | 41 (5.3) | 44 (5.6) |
| **Parity (N (%))** | | | | | | |
| Primiparous | 260 (45.1) | 257 (44.2) | 266 (44.9%) | 265 (44.1%) | 338 (43.8) | 336 (42.9) |
| Multiparous | 317 (54.9) | 324 (55.8) | 327 (55.1%) | 336 (55.9%) | 433 (56.2) | 447 (57.1) |
| **Age (N (%))** |  |  |  |  |  |  |
| <25 years | 97 (16.8) | 85 (14.6) | 100 (16.9%) | 90 (15%) | 125 (17.3) | 116 (15.7) |
| 25 to 29 years | 141 (24.4) | 165 (28.4) | 147 (24.8%) | 169 (28.1%) | 199 (27.6) | 215 (29.1) |
| 30 to 34 years | 187 (32.4) | 174 (29.9) | 188 (31.7%) | 182 (30.3%) | 192 (26.6) | 205 (27.8) |
| ≥35 years | 152 (26.3) | 157 (27) | 158 (26.6%) | 160 (26.6%) | 206 (28.5) | 202 (27.4) |
| **Centre (N (%))** | | | | | | |
| Bradford | 19 (3.3) | 22 (3.8) | 25 (4.2%) | 28 (4.7%) | 25 (3.2) | 28 (3.6) |
| Glasgow | 130 (22.5) | 132 (22.7) | 131 (22.1%) | 134 (22.3%) | 131 (17.0) | 134 (17.1) |
| Manchester | 67 (11.6) | 67 (11.5) | 70 (11.8%) | 69 (11.5%) | 70 (9.1) | 69 (8.8) |
| Newcastle | 120 (20.8) | 116 (20) | 122 (20.6%) | 120 (20%) | 122 (15.8) | 120 (15.3) |
| Sunderland | 0 | 0 | 0 | 0 | 41 (5.3) | 43 (5.5) |
| St George’s, London | 53 (9.2) | 55 (9.5) | 54 (9.1%) | 57 (9.5%) | 54 (7.0) | 57 (7.3) |
| St Thomas’s, London | 188 (32.6) | 189 (32.5) | 191 (32.2%) | 193 (32.1%) | 191 (24.8) | 193 (24.6) |
| King’s College Hospital, London | 0 | 0 | 0 | 0 | 137 (17.8) | 139 (17.8) |

**Table 1: continued**

| **First clinic** | | | | | | |
| --- | --- | --- | --- | --- | --- | --- |
| N (%) | 538 (93.2) | 545 (93.8) | 593 (100.0) | 601 (100.0) | 771 (100.0) | 783 (100.0) |
| Median (IQR) gestation (weeks) | 17 (16.1, 17.9) | 17 (16.1, 18.0) | 17.0 (16.1, 17.9) | 17.0 (16.1, 18.0) | 17 (16.1, 18.0) | 17.0  (16.1, 18.0) |
| **Second clinic** | | | | | | |
| N (%) | 500 (86.7) | 477 (82.1) | 591 (99.7) | 598 (99.5) | 768 (99.6) | 780 (99.6) |
| Median (IQR) gestation (weeks) | 27.7 (27.3, 28.1) | 27.7  (27.3, 28.1) | 27.7 (27.3, 28.3) | 27.7 (27.3, 28.1) | 27.7  (27.3, 28.3) | 27.7  (27.3, 28.3) |
| **Third clinic** | | | | | | |
| N (%) | 407 (70.5) | 374 (64.4) | 524 (88.4) | 485 (80.7) | 662 (85.9) | 630 (80.5) |
| Median (IQR) gestation (weeks) | 34.7 (34.3, 35.1) | 34.6  (34.3, 35.1) | 34.7 (34.3, 35.3) | 34.7 (34.3, 35.3) | 34.8 (34.3, 35.3) | 34.7 (34.3, 35.4) |

*****The 1158 participants whose results are in the first two columns are a subgroup of the 1194 whose results are presented in the middle two columns who are in turn a subgroup of the 1554 whose results are in the final two columns.

N: number; IQR: Interquartile range

**Table S2: NMR metabolic measures**

| **Molecular class** | **Lipid, lipoprotein or metabolite name** | **Units*** |
| --- | --- | --- |
|  |  |  |
| **Extremely large VLDL** | Concentration of chylomicrons and extremely large VLDL particles | mol/l |
|  | Total lipids in chylomicrons and extremely large VLDL | mmol/l |
|  | Phospholipids in chylomicrons and extremely large VLDL | mmol/l |
|  | Total cholesterol in chylomicrons and extremely large VLDL | mmol/l |
|  | Free cholesterol in chylomicrons and extremely large VLDL | mmol/l |
|  | Triglycerides in chylomicrons and extremely large VLDL | mmol/l |
| **Very large VLDL** | Concentration of very large VLDL particles | mol/l |
|  | Total lipids in very large VLDL | mmol/l |
|  | Phospholipids in very large VLDL | mmol/l |
|  | Total cholesterol in very large VLDL | mmol/l |
|  | Cholesterol esters in very large VLDL | mmol/l |
|  | Free cholesterol in very large VLDL | mmol/l |
|  | Triglycerides in very large VLDL | mmol/l |
| **Large VLDL** | Concentration of large VLDL particles | mol/l |
|  | Total lipids in large VLDL | mmol/l |
|  | Phospholipids in large VLDL | mmol/l |
|  | Total cholesterol in large VLDL | mmol/l l |
|  | Cholesterol esters in large VLDL | mmol/l |
|  | Free cholesterol in large VLDL | mmol/l |
|  | Triglycerides in large VLDL | mmol/l |
| **Medium VLDL** | Concentration of large VLDL particles | mol/l |
|  | Total lipids in small VLDL | mmol/l |
|  | Phospholipids in small VLDL | mmol/l |
|  | Total cholesterol in small VLDL | mmol/l |
|  | Cholesterol esters in small VLDL | mmol/l |
|  | Free cholesterol in small VLDL | mmol/l |
|  | Triglycerides in small VLDL | mmol/l |
| **Small VLDL** | Concentration of very small VLDL particles | mol/l |
|  | Total lipids in very small VLDL | mmol/l |
|  | Phospholipids in very small VLDL | mmol/l |
|  | Total cholesterol in very small VLDL | mmol/l |
|  | Cholesterol esters in very small VLDL | mmol/l |
|  | Free cholesterol in very small VLDL | mmol/l |
|  | Triglycerides in very small VLDL | mmol/l |
| **IDI** | Concentration of IDL particles | mol/l |
|  | Total lipids in IDL | mmol/l |
|  | Phospholipids in IDL | mmol/l |
|  | Total cholesterol in IDL | mmol/l |
|  | Cholesterol esters in IDL | mmol/l |
|  | Free cholesterol in IDL | mmol/l |
|  | Triglycerides in IDL | mmol/l |
| **Large LDL** | Concentration of large LDL particles | mol/l |
|  | Total lipids in large LDL | mmol/l |
|  | Phospholipids in large LDL | mmol/l |
|  | Total cholesterol in large LDL | mmol/l |
|  | Cholesterol esters in large LDL | mmol/l |
|  | Free cholesterol in large LDL | mmol/l |
|  | Triglycerides in large LDL | mmol/l |

**Table S2: NMR metabolic profiles continued**

| **Molecular class** | **Lipid, lipoprotein or metabolite name** | **Units*** |
| --- | --- | --- |
|  |  |  |
| **Medium LDL** | Concentration of medium LDL particles | mol/l |
|  | Total lipids in medium LDL | mmol/l |
|  | Phospholipids in medium LDL | mmol/l |
|  | Total cholesterol in medium LDL | mmol/l |
|  | Cholesterol esters in medium LDL | mmol/l |
|  | Free cholesterol in medium LDL | mmol/l |
|  | Triglycerides in medium LDL | mmol/l |
| **Small LDL** | Concentration of small LDL particles | mol/l |
|  | Total lipids in small LDL | mmol/l |
|  | Phospholipids in small LDL | mmol/l |
|  | Total cholesterol in small LDL | mmol/l |
|  | Cholesterol esters in small LDL | mmol/l |
|  | Free cholesterol in small LDL | mmol/l |
|  | Triglycerides in small LDL | mmol/l |
| **Very large HDL** | Concentration of very large HDL particles | mol/l |
|  | Total lipids in very large HDL | mmol/l |
|  | Phospholipids in very large HDL | mmol/l |
|  | Total cholesterol in very large HDL | mmol/l |
|  | Cholesterol esters in very large HDL | mmol/l |
|  | Free cholesterol in very large HDL | mmol/l |
|  | Triglycerides in very large HDL | mmol/l |
| **Large HDL** | Concentration of large HDL particles | mol/l |
|  | Total lipids in large HDL | mmol/l |
|  | Phospholipids in large HDL | mmol/l |
|  | Total cholesterol in large HDL | mmol/l |
|  | Cholesterol esters in large HDL | mmol/l |
|  | Free cholesterol in large HDL | mmol/l |
|  | Triglycerides in large HDL | mmol/l |
| **Medium HDL** | Concentration of medium HDL particles | mol/l |
|  | Total lipids in medium HDL | mmol/l |
|  | Phospholipids in medium HDL | mmol/l |
|  | Total cholesterol in medium HDL | mmol/l |
|  | Cholesterol esters in medium HDL | mmol/l |
|  | Free cholesterol in medium HDL | mmol/l |
|  | Triglycerides in medium HDL | mmol/l |
| **Small HDL** | Concentration of small HDL particles | mol/l |
|  | Total lipids in small HDL | mmol/l |
|  | Phospholipids in small HDL | mmol/l |
|  | Total cholesterol in small HDL | mmol/l |
|  | Cholesterol esters in small HDL | mmol/l |
|  | Free cholesterol in small HDL | mmol/l |
|  | Triglycerides in small HDL | mmol/l |
| **Lipoprotein particle size** | Mean diameter for VLDL particles | nm |
|  | Mean diameter for LDL particles | nm |
|  | Mean diameter for HDL particles | nm |

**Table S2: NMR metabolic profiles continued**

| **Molecular class** | **Lipid, lipoprotein or metabolite name** | **Units*** |
| --- | --- | --- |
|  |  |  |
| **Cholesterol concentrations** | Total cholesterol | mmol/l |
|  | Total cholesterol in VLDL | mmol/l |
|  | Remnant cholesterol (non-HDL and non-LDL cholesterol) | mmol/l |
|  | Total cholesterol in LDL | mmol/l |
|  | Total cholesterol in HDL | mmol/l |
|  | Total cholesterol in HDL2 | mmol/l |
|  | Total cholesterol in HDL3 | mmol/l |
|  | Esterified cholesterol | mmol/l |
|  | Free cholesterol | mmol/l |
| **Glycerides and phospholipid concentrations (and one ratio)** | Total triglycerides | mmol/l |
|  | Triglycerides in VLDL | mmol/l |
|  | Triglycerides in LDL | mmol/l |
|  | Triglycerides in HDL | mmol/l |
|  | Total phosphoglycerides | mmol/l |
|  | Ratio of triglycerides to phosphoglycerides |  |
|  | Phosphatydilcholine and other cholines | mmol/l |
|  | Sphingomyelins | mmol/l |
|  | Total cholines | mmol/l |
| **Apolipoprotein concentrations (and one ratio)** | Apolipoprotein A-1 | g/l |
|  | Apolipoprotein B | g/l |
|  | Ratio of apolipoprotein B to apolipoprotein A-1 |  |
| **Fatty acid concentrations** | Total fatty acids | mmol/l |
|  | Estimated degree of saturation |  |
|  | 22:6, docosahexaenoic acid | mmol/l |
|  | 18:2 linoleic acid | mmol/l |
|  | Omega-3 fatty acids | mmol/l |
|  | Omega-6 fatty acids | mmol/l |
|  | Polyunsaturated fatty acids | mmol/l |
|  | Monounsaturated fatty acids; 16:1, 18:1 | mmol/l |
|  | Saturated fatty acids | mmol/l |
| **Fatty acid ratios** | Ratio of 22:6, docosahexaenoic acid to total fatty acids | % |
|  | Ratio of 18:2 linoleic acid to total fatty acids | % |
|  | Ratio of omega-3 fatty acids to total fatty acids | % |
|  | Ratio of omega-6 fatty acids to total fatty acids | % |
|  | Ratio of polyunsaturated fatty acids to total fatty acids | % |
|  | Ratio of monounsaturated fatty acids to total fatty acids | % |
|  | Ratio of saturated fatty acids to total fatty acids | % |
| **Glycolysis related metabolite** | Glucose | mmol/l |
|  | Lactate | mmol/l |
|  | Pyruvate | mmol/l |
|  | Citrate | mmol/l |
|  | Glycerol | mmol/l |

**Table S2: NMR metabolic profiles continued**

| **Molecular class** | **Lipid, lipoprotein or metabolite name** | **Units*** |
| --- | --- | --- |
|  |  |  |
| **Amino acid concentrations** | Alanine | mmol/l |
|  | Glutamine | mmol/l |
|  | Glycine | mmol/l |
|  | Histidine | mmol/l |
| branched | Isoleucine | mmol/l |
| branched | Leucine | mmol/l |
| branched | Valine | mmol/l |
| aromatic | Phenylalanine | mmol/l |
| aromatic | Tyrosine | mmol/l |
| **Ketone body concentrations** | Acetate | mmol/l |
|  | Acetoacetate | mmol/l |
|  | 3-hydroxybutyrate | mmol/l |
| **Fluid balance marker** | Albumin | mmol/l |
|  | Creatinine | mmol/l |
| **Inflammation marker** | Glycoprotein acetyls, mainly a1-acid glycoprotein | mmol/l |

* These are the units used throughout the paper for each of the metabolic measures, unless we state that we are presenting results in standard deviation (SD) units. Where we present results that are the mean (in control participants) at 16-weeks these are the units. Where we present change in metabolic marker (between 16- to 36-weeks) or difference in change of metabolic markers the units are those listed in the table above per one week of gestational age.

VLDL: very low density lipoprotein; LDL: low density lipoprotein; IDL: intermediate density lipoprotein; HDL: high density lipoprotein

## Table S3: Absolute difference between 16- and 36-weeks of gestation for each metabolic trait in obese pregnant women who were randomised to the control arm of the UPBEAT RCT (N = 577). NOTE: ONLY THE FINALCOLUMN OF RESULTS HAS CHANGED. The whole table is replaced because the statistical package produces the whole table

|  | Mean absolute difference between 16 and 36 weeks of gestation in original units* (95% CI) | Mean absolute difference between 16 and 36 weeks of gestational age in SD units^$^ |
| --- | --- | --- |
| **Extremely large VLDL** | | |
| Concentration of chylomicrons and extremely large VLDL particles (mol/l) | 1.1434x10^-10^  (1.1003x10^-10^ , 1.1864x10^-10^ ) | 1.4744 (1.3386, 1.6101) |
| Total lipids in chylomicrons and extremely large VLDL (mmol/l) | 0.0253 (0.0243, 0.0262) | 1.5209 (1.3852, 1.6567) |
| Phospholipids in chylomicrons and extremely large VLDL (mmol/l) | 0.0038 (0.0037, 0.0039) | 1.7572 (1.6199, 1.8946) |
| Total cholesterol in chylomicrons and extremely large VLDL (mmol/l) | 0.0058 (0.0056, 0.0059) | 1.9934 (1.8573, 2.1295) |
| Cholesterol esters in chylomicrons and extremely large VLDL (mmol/l) | 0.0031 (0.0030, 0.0032) | 1.9763 (1.8428, 2.1099) |
| Free cholesterol in chylomicrons and extremely large VLDL (mmol/l) | 0.0026 (0.0026, 0.0027) | 1.8955 (1.7569, 2.0341) |
| Triglycerides in chylomicrons and extremely large VLDL (mmol/l) | 0.0157 (0.0150, 0.0163) | 1.3390 (1.2028, 1.4753) |
| **Very large VLDL** | | |
| Concentration of very large VLDL particles (mol/l) | 8.5288x10^-10^  (8.2336x10^-10^ , 8.8240x10^-10^ ) | 1.8215 (1.6907, 1.9523) |
| Total lipids in very large VLDL (mmol/l) | 0.0834 (0.0805, 0.0862) | 1.8329 (1.7022, 1.9636) |
| Phospholipids in very large VLDL (mmol/l) | 0.0147 (0.0142, 0.0152) | 1.9244 (1.7932, 2.0556) |
| Total cholesterol in very large VLDL (mmol/l) | 0.0164 (0.0159, 0.0169) | 1.8729 (1.7442, 2.0016) |
| Cholesterol esters in very large VLDL (mmol/l) | 0.0084 (0.0081, 0.0086) | 1.7910 (1.6643, 1.9177) |
| Free cholesterol in very large VLDL (mmol/l) | 0.0080 (0.0078, 0.0083) | 1.9491 (1.8180, 2.0803) |
| Triglycerides in very large VLDL (mmol/l) | 0.0523 (0.0504, 0.0542) | 1.7856 (1.6544, 1.9168) |

**Table S3: continued**

| **Large VLDL** | | |
| --- | --- | --- |
| Concentration of large VLDL particles (mol/l) | 5.2624x10^-9^  (5.0853x10^-9^, 5.4396x10^-9^) | 1.9810 (1.8515, 2.1106) |
| Total lipids in large VLDL (mmol/l) | 0.3066 (0.2964, 0.3168) | 1.9925 (1.8630, 2.1220) |
| Phospholipids in large VLDL (mmol/l) | 0.0588 (0.0569, 0.0607) | 2.1000 (1.9692, 2.2308) |
| Total cholesterol in large VLDL (mmol/l) | 0.0688 (0.0667, 0.0710) | 2.0026 (1.8759, 2.1293) |
| Cholesterol esters in large VLDL (mmol/l) | 0.0310 (0.0300, 0.0319) | 1.8446 (1.7224, 1.9668) |
| Free cholesterol in large VLDL (mmol/l) | 0.0379 (0.0367, 0.0391) | 2.1227 (1.9906, 2.2549) |
| Triglycerides in large VLDL (mmol/l) | 0.1790 (0.1727, 0.1852) | 1.9476 (1.8173, 2.0779) |
| **Medium VLDL** | | |
| Concentration of medium VLDL particles (mol/l) | 1.4126x10^-8^  (1.3685x10^-8^, 1.4566x10^-8^) | 2.0533 (1.9282, 2.1784) |
| Total lipids in medium VLDL (mmol/l) | 0.4744 (0.4598, 0.4889) | 2.0810 (1.9556, 2.2063) |
| Phospholipids in medium VLDL (mmol/l) | 0.0946 (0.0917, 0.0974) | 2.1388 (2.0131, 2.2644) |
| Total cholesterol in medium VLDL (mmol/l) | 0.1270 (0.1234, 0.1306) | 2.2276 (2.1005, 2.3546) |
| Cholesterol esters in medium VLDL (mmol/l) | 0.0619 (0.0601, 0.0637) | 2.1050 (1.9786, 2.2314) |
| Free cholesterol in medium VLDL (mmol/l) | 0.0651 (0.0632, 0.0670) | 2.2462 (2.1169, 2.3756) |
| Triglycerides in medium VLDL (mmol/l) | 0.2528 (0.2444, 0.2612) | 1.9532 (1.8282, 2.0781) |
| **Small VLDL** | | |
| Concentration of small VLDL particles (mol/l) | 1.9052x10^-8^  (1.8499x10^-8^, 1.9605x10^-8^) | 2.3518 (2.2210, 2.4827) |
| Total lipids in small VLDL (mmol/l) | 0.3694 (0.3587, 0.3801) | 2.3770 (2.2453, 2.5087) |
| Phospholipids in small VLDL (mmol/l) | 0.0777 (0.0754, 0.0799) | 2.3103 (2.1823, 2.4383) |
| Total cholesterol in small VLDL (mmol/l) | 0.1224 (0.1185, 0.1263) | 2.2717 (2.1356, 2.4078) |
| Cholesterol esters in small VLDL (mmol/l) | 0.0672 (0.0648, 0.0697) | 1.9488 (1.8153, 2.0822) |
| Free cholesterol in small VLDL (mmol/l) | 0.0552 (0.0536, 0.0568) | 2.5385 (2.4013, 2.6757) |
| Triglycerides in small VLDL (mmol/l) | 0.1692 (0.1640, 0.1744) | 2.2049 (2.0758, 2.3339) |

**Table S3: continued**

| **Very small VLDL** | | |
| --- | --- | --- |
| Concentration of very small VLDL particles (mol/l) | 1.8605x10^-8^  (1.7963x10^-8^, 1.9247x10^-8^) | 2.2794 (2.1422, 2.4166) |
| Total lipids in very small VLDL (mmol/l) | 0.2258 (0.2177, 0.2338) | 2.1825 (2.0466, 2.3184) |
| Phospholipids in very small VLDL (mmol/l) | 0.0638 (0.0612, 0.0664) | 1.8884 (1.7570, 2.0198) |
| Total cholesterol in very small VLDL (mmol/l) | 0.0845 (0.0809, 0.0881) | 1.6930 (1.5632, 1.8227) |
| Cholesterol esters in very small VLDL (mmol/l) | 0.0490 (0.0467, 0.0512) | 1.5036 (1.3759, 1.6312) |
| Free cholesterol in very small VLDL (mmol/l) | 0.0355 (0.0340, 0.0369) | 1.9718 (1.8349, 2.1087) |
| Triglycerides in very small VLDL (mmol/l) | 0.0775 (0.0751, 0.0799) | 2.6855 (2.5380, 2.8330) |
| **IDL** | | |
| Concentration of IDL particles (mol/l) | 3.8023x10^-8^  (3.6572x10^-8^, 3.9474x10^-8^) | 1.7353 (1.6148, 1.8558) |
| Total lipids in IDL (mmol/l) | 0.3651 (0.3506, 0.3796) | 1.6281 (1.5094, 1.7467) |
| Phospholipids in IDL (mmol/l) | 0.0761 (0.0725, 0.0798) | 1.3530 (1.2370, 1.4689) |
| Total cholesterol in IDL (mmol/l) | 0.2056 (0.1966, 0.2146) | 1.3939 (1.2797, 1.5081) |
| Cholesterol esters in IDL (mmol/l) | 0.1525 (0.1463, 0.1588) | 1.4720 (1.3576, 1.5865) |
| Free cholesterol in IDL (mmol/l) | 0.0531 (0.0503, 0.0559) | 1.1812 (1.0678, 1.2946) |
| Triglycerides in IDL (mmol/l) | 0.0833 (0.0806, 0.0859) | 2.8347 (2.6825, 2.9870) |
| **Large LDL** | | |
| Concentration of large LDL particles (mol/l) | 6.3247x10^-8^  (6.0733x10^-8^, 6.5762x10^-8^) | 1.6580 (1.5392, 1.7769) |
| Total lipids in large LDL (mmol/l) | 0.4301 (0.4124, 0.4479) | 1.5768 (1.4593, 1.6943) |
| Phospholipids in large LDL (mmol/l) | 0.0808 (0.0774, 0.0843) | 1.4571 (1.3421, 1.5722) |
| Total cholesterol in large LDL (mmol/l) | 0.2782 (0.2656, 0.2908) | 1.4009 (1.2864, 1.5154) |
| Cholesterol esters in large LDL (mmol/l) | 0.2182 (0.2087, 0.2277) | 1.4597 (1.3448, 1.5747) |
| Free cholesterol in large LDL (mmol/l) | 0.0600 (0.0569, 0.0631) | 1.2054 (1.0923, 1.3186) |
| Triglycerides in large LDL (mmol/l) | 0.0711 (0.0690, 0.0732) | 2.7454 (2.6016, 2.8893) |

**Table S3: continued**

| **Medium LDL** | | |
| --- | --- | --- |
| Concentration of medium LDL particles (mol/l) | 5.4777x10^-8^  (5.2623x10^-8^, 5.6932x10^-8^) | 1.6759 (1.5578, 1.7941) |
| Total lipids in medium LDL (mmol/l) | 0.2677 (0.2570, 0.2785) | 1.6221 (1.5051, 1.7392) |
| Phospholipids in medium LDL (mmol/l) | 0.0562 (0.0543, 0.0582) | 1.7851 (1.6702, 1.9000) |
| Total cholesterol in medium LDL (mmol/l) | 0.1768 (0.1688, 0.1848) | 1.4073 (1.2929, 1.5218) |
| Cholesterol esters in medium LDL (mmol/l) | 0.1417 (0.1351, 0.1483) | 1.3726 (1.2578, 1.4875) |
| Free cholesterol in medium LDL (mmol/l) | 0.0351 (0.0337, 0.0365) | 1.5349 (1.4224, 1.6473) |
| Triglycerides in medium LDL (mmol/l) | 0.0347 (0.0337, 0.0357) | 2.7407 (2.5984, 2.8831) |
| **Small LDL** | | |
| Concentration of small LDL particles (mol/l) | 6.0152x10^-8^  (5.7904x10^-8^, 6.2400x10^-8^) | 1.6958 (1.5801, 1.8115) |
| Total lipids in small LDL (mmol/l) | 0.1629 (0.1566, 0.1691) | 1.6348 (1.5200, 1.7496) |
| Phospholipids in small LDL (mmol/l) | 0.0368 (0.0357, 0.0379) | 1.8173 (1.7069, 1.9276) |
| Total cholesterol in small LDL (mmol/l) | 0.1036 (0.0988, 0.1083) | 1.3643 (1.2513, 1.4773) |
| Cholesterol esters in small LDL (mmol/l) | 0.0817 (0.0778, 0.0857) | 1.3012 (1.1879, 1.4144) |
| Free cholesterol in small LDL (mmol/l) | 0.0218 (0.0211, 0.0226) | 1.5944 (1.4836, 1.7053) |
| Triglycerides in small LDL (mmol/l) | 0.0226 (0.0220, 0.0232) | 2.8622 (2.7234, 3.0009) |
| **Very large HDL** | | |
| Concentration of very large HDL particles (mol/l) | 7.9744x10^-8^  (7.6882x10^-8^, 8.2605x10^-8^) | 0.4226 (0.3545, 0.4906) |
| Total lipids in very large HDL (mmol/l) | 0.0778 (0.0749, 0.0808) | 0.4068 (0.3384, 0.4752) |
| Phospholipids in very large HDL (mmol/l) | 0.0240 (0.0227, 0.0252) | 0.2372 (0.1692, 0.3052) |
| Total cholesterol in very large HDL (mmol/l) | 0.0385 (0.0369, 0.0401) | 0.4448 (0.3733, 0.5162) |
| Cholesterol esters in very large HDL (mmol/l) | 0.0285 (0.0273, 0.0297) | 0.4683 (0.3970, 0.5396) |
| Free cholesterol in very large HDL (mmol/l) | 0.0100 (0.0095, 0.0104) | 0.3850 (0.3122, 0.4577) |
| Triglycerides in very large HDL (mmol/l) | 0.0152 (0.0149, 0.0155) | 2.0577 (1.9505, 2.1650) |

**Table S3: continued**

| **Large HDL** | | |
| --- | --- | --- |
| Concentration of large HDL particles (mol/l) | 1.0288x10^-8^  (1.5883x10^-9^, 1.8988x10^-8^) | 0.0250 (-0.0529, 0.1030) |
| Total lipids in large HDL (mmol/l) | -0.0024 (-0.0079, 0.0031) | -0.0073 (-0.0849, 0.0702) |
| Phospholipids in large HDL (mmol/l) | -0.0096 (-0.0121, -0.0071) | -0.0791 (-0.1615, 0.0033) |
| Total cholesterol in large HDL (mmol/l) | -0.0142 (-0.0170, -0.0113) | -0.0948 (-0.1694, -0.0203) |
| Cholesterol esters in large HDL (mmol/l) | -0.0091 (-0.0113, -0.0069) | -0.0798 (-0.1549, -0.0048) |
| Free cholesterol in large HDL (mmol/l) | -0.0051 (-0.0057, -0.0044) | -0.1428 (-0.2160, -0.0695) |
| Triglycerides in large HDL (mmol/l) | 0.0213 (0.0208, 0.0217) | 1.4396 (1.3464, 1.5327) |
| **Medium HDL** | | |
| Concentration of medium HDL particles (mol/l) | -1.7335x10^-7^  (-1.8439x10^-7^, -1.6231x10^-7^) | -0.5444 (-0.6467, -0.4420) |
| Total lipids in medium HDL (mmol/l) | -0.0826 (-0.0874, -0.0777) | -0.6007 (-0.7040, -0.4974) |
| Phospholipids in medium HDL (mmol/l) | -0.0184 (-0.0204, -0.0163) | -0.2986 (-0.4004, -0.1967) |
| Total cholesterol in medium HDL (mmol/l) | -0.0769 (-0.0799, -0.0739) | -1.0402 (-1.1484, -0.9319) |
| Cholesterol esters in medium HDL (mmol/l) | -0.0682 (-0.0707, -0.0657) | -1.1619 (-1.2712, -1.0527) |
| Free cholesterol in medium HDL (mmol/l) | -0.0086 (-0.0092, -0.0081) | -0.5468 (-0.6521, -0.4414) |
| Triglycerides in medium HDL (mmol/l) | 0.0126 (0.0124, 0.0129) | 1.2199 (1.1251, 1.3147) |
| **Small HDL** | | |
| Concentration of small HDL particles (mol/l) | -1.9756x10^-9^  (-1.4709x10^-8^, 1.0758x10^-8^) | -0.0017 (-0.0880, 0.0846) |
| Total lipids in small HDL (mmol/l) | -0.0057 (-0.0086, -0.0028) | -0.0531 (-0.1412, 0.0350) |
| Phospholipids in small HDL (mmol/l) | -0.0324 (-0.0349, -0.0298) | -0.4458 (-0.5332, -0.3584) |
| Total cholesterol in small HDL (mmol/l) | 0.0035 (0.0016, 0.0054) | 0.0707 (-0.0361, 0.1775) |
| Cholesterol esters in small HDL (mmol/l) | 0.0044 (0.0024, 0.0064) | 0.0937 (-0.0150, 0.2023) |
| Free cholesterol in small HDL (mmol/l) | -0.0010 (-0.0014, -0.0007) | -0.0884 (-0.1822, 0.0054) |
| Triglycerides in small HDL (mmol/l) | 0.0236 (0.0231, 0.0242) | 2.1177 (2.0032, 2.2322) |

**Table S3: continued**

| **Lipoprotein particle size** | | |
| --- | --- | --- |
| Mean diameter for VLDL particles (mm) | 0.9280 (0.9079, 0.9481) | 0.8301 (0.7538, 0.9064) |
| Mean diameter for LDL particles (mm) | -0.0224 (-0.0229, -0.0218) | -0.3429 (-0.4197, -0.2660) |
| Mean diameter for HDL particles (mm) | 0.0193 (0.0165, 0.0222) | 0.1039 (0.0386, 0.1691) |
| **Cholesterol** | | |
| Serum total cholesterol (mmol/l) | 1.1410 (1.1005, 1.1815) | 1.5301 (1.4169, 1.6433) |
| Total cholesterol in VLDL (mmol/l) | 0.4257 (0.4140, 0.4375) | 2.3951 (2.2656, 2.5245) |
| Remnant cholesterol (non-HDL, non-LDL -cholesterol) (mmol/l) | 0.6311 (0.6117, 0.6504) | 2.1657 (2.0402, 2.2912) |
| Total cholesterol in LDL (mmol/l) | 0.5585 (0.5332, 0.5838) | 1.3984 (1.2842, 1.5126) |
| Total cholesterol in HDL (mmol/l) | -0.0495 (-0.0558, -0.0432) | -0.1836 (-0.2713, -0.0958) |
| Total cholesterol in HDL2 (mmol/l) | -0.0933 (-0.0994, -0.0873) | -0.3804 (-0.4696, -0.2912) |
| Total cholesterol in HDL3 (mmol/l) | 0.0436 (0.0427, 0.0445) | 1.4894 (1.3966, 1.5823) |
| Esterified cholesterol (mmol/l) | 0.7862 (0.7591, 0.8134) | 1.3998 (1.2973, 1.5022) |
| Free cholesterol (mmol/l) | 0.3427 (0.3306, 0.3547) | 1.4004 (1.2667, 1.5342) |
| **Glycerides and phospholipids** | | |
| Serum total triglycerides (mmol/l) | 1.0325 (1.0030, 1.0621) | 2.4433 (2.3118, 2.5749) |
| Triglycerides in VLDL (mmol/l) | 0.7475 (0.7238, 0.7712) | 2.0889 (1.9625, 2.2154) |
| Triglycerides in LDL (mmol/l) | 0.1284 (0.1247, 0.1321) | 2.7944 (2.6514, 2.9375) |
| Triglycerides in HDL (mmol/l) | 0.0726 (0.0711, 0.0740) | 2.2624 (2.1419, 2.3829) |
| Total phosphoglycerides (mmol/l) | 0.4613 (0.4535, 0.4691) | 1.5492 (1.4272, 1.6711) |
| Ratio of triglycerides to phosphoglycerides (mmol/l) | 0.2263 (0.2214, 0.2312) | 1.4034 (1.3131, 1.4936) |
| Phosphatidylcholine and other cholines (mmol/l) | 0.4833 (0.4742, 0.4924) | 1.6998 (1.5741, 1.8255) |
| Sphingomyelins (mmol/l) | 0.0411 (0.0388, 0.0434) | 0.5664 (0.4357, 0.6970) |
| Total cholines (mmol/l) | 0.4688 (0.4592, 0.4783) | 1.4979 (1.3543, 1.6414) |
| **Apolipoproteins** | | |
| Apolipoprotein A-I (g/l) | 0.0886 (0.0850, 0.0921) | 0.5564 (0.4660, 0.6468) |
| Apolipoprotein B (g/l) | 0.3360 (0.3260, 0.3460) | 2.2789 (2.1537, 2.4041) |
| **Apolipoproteins ratio** | | |
| Ratio of apolipoprotein B to apolipoprotein A-I | 0.1627 (0.1574, 0.1680) | 1.9077 (1.7944, 2.0209) |
| **Fatty acids** |  |  |
| Total fatty acids (mmol/l) | 3.8748 (3.7979, 3.9517) | 1.9642 (1.8277, 2.1006) |
| Estimated degree of unsaturation | -0.0371 (-0.0376, -0.0366) | -0.8438 (-0.9304, -0.7571) |
| 22:6, docosahexaenoic acid (mmol/l) | 0.0137 (0.0130, 0.0143) | 0.2957 (0.1934, 0.3981) |
| 18:2, linoleic acid (mmol/l) | 0.9050 (0.8813, 0.9288) | 1.5801 (1.4440, 1.7161) |
| Omega-3 fatty acids (mmol/l) | 0.0907 (0.0889, 0.0924) | 0.6754 (0.5763, 0.7744) |
| Omega-6 fatty acids (mmol/l) | 0.8278 (0.8035, 0.8521) | 1.3989 (1.2596, 1.5382) |
| Polyunsaturated fatty acids (mmol/l) | 0.9185 (0.8924, 0.9447) | 1.3271 (1.1918, 1.4623) |
| Monounsaturated fatty acids; 16:1, 18:1 (mmol/l) | 1.4682 (1.4409, 1.4956) | 2.0734 (1.9541, 2.1927) |
| Saturated fatty acids (mmol/l) | 1.4802 (1.4515, 1.5089) | 1.9564 (1.8160, 2.0968) |
| **Fatty acid ratios** | | |
| Ratio of 22:6 docosahexaenoic acid to total fatty acids (%) | -0.3120 (-0.3277, -0.2963) | -0.7954 (-0.8618, -0.7289) |
| Ratio of 18:2 linoleic acid to total fatty acids (%) | -0.4925 (-0.5183, -0.4668) | -0.1792 (-0.2577, -0.1008) |
| Ratio of omega-3 fatty acids to total fatty acids (%) | -0.5671 (-0.5824, -0.5519) | -0.7898 (-0.8608, -0.7187) |
| Ratio of omega-6 fatty acids to total fatty acids (%) | -2.0000 (-2.0344, -1.9657) | -0.8198 (-0.9013, -0.7382) |
| Ratio of polyunsaturated fatty acids to total fatty acids (%) | -2.5647 (-2.6054, -2.5240) | -0.9330 (-1.0110, -0.8549) |
| Ratio of monounsaturated fatty acids to total fatty acids (%) | 2.1382 (2.0245, 2.2519) | 0.6084 (0.5437, 0.6732) |
| Ratio of saturated fatty acids to total fatty acids (%) | 0.2234 (0.2111, 0.2357) | 0.1714 (0.0729, 0.2699) |
| **Glycolysis related metabolites** | | |
| Glucose (mmol/l) | 0.3700 (0.3506, 0.3894) | 0.5445 (0.4015, 0.6875) |
| Lactate (mmol/l) | 0.1899 (0.1757, 0.2041) | 0.3612 (0.2668, 0.4555) |
| Pyruvate (mmol/l) | 0.0248 (0.0241, 0.0255) | 0.5726 (0.4623, 0.6828) |
| Citrate (mmol/l) | 0.0180 (0.0174, 0.0186) | 1.0796 (0.9674, 1.1917) |
| Glycerol (mmol/l) | -0.0055 (-0.0060, -0.0051) | -0.1979 (-0.2940, -0.1019) |
| **Amino acids** | | |
| Alanine (mmol/l) | 0.0329 (0.0322, 0.0335) | 0.8250 (0.7138, 0.9362) |
| Glutamine (mmol/l) | -0.0030 (-0.0036, -0.0023) | -0.0686 (-0.1589, 0.0218) |
| Glycine (mmol/l) | 0.0093 (0.0089, 0.0096) | 0.3764 (0.2871, 0.4656) |
| Histidine (mmol/l) | 0.0014 (0.0014, 0.0015) | 0.1996 (0.0925, 0.3066) |
| **Amino acids - branched chain** | | |
| Isoleucine (mmol/l) | 0.0037 (0.0032, 0.0042) | 0.2880 (0.1863, 0.3897) |
| Leucine (mmol/l) | 1.3926x10^-5^  (-0.0003, 0.0003) | 0.0059 (-0.0895, 0.1013) |
| Valine (mmol/l) | -0.0207 (-0.0218, -0.0196) | -0.6803 (-0.7740, -0.5866) |
| **Amino acids - aromatic** | | |
| Phenylalanine (mmol/l) | 0.0058 (0.0057, 0.0059) | 0.5802 (0.4807, 0.6797) |
| Tyrosine (mmol/l) | -0.0013 (-0.0014, -0.0012) | -0.1398 (-0.2356, -0.0441) |
| **Ketone bodies** | | |
| Acetate (mmol/l) | 0.0007 (0.0007, 0.0008) | 0.1013 (-0.0371, 0.2398) |
| Acetoacetate (mmol/l) | 0.0017 (0.0012, 0.0021) | 0.1424 (0.0378, 0.2470) |
| 3-hydroxybutyrate (mmol/l) | 0.0216 (0.0185, 0.0248) | 0.3319 (0.2354, 0.4285) |
| **Fluid balance** | | |
| Creatinine (mmol/l) | 0.0011 (0.0011, 0.0012) | 0.1847 (0.0758, 0.2936) |
| Albumin (mmol/l) | -0.0029 (-0.0029, -0.0028) | -0.8731 (-0.9871, -0.7591) |
| **Inflammation** | | |
| Glycoprotein acetyls, mainly a1-acid glycoprotein (mmol/l) | 0.2370 (0.2333, 0.2406) | 1.7367 (1.6290, 1.8444) |

* Total difference in each trait between 16 and 36 weeks in original units as given in first column

^$^ Total difference in each trait between 16 and 36 weeks in SD units; for these analyses the SD for each trait at 16 weeks was used

VLDL: very low density lipoprotein; LDL: low density lipoprotein; IDL: intermediate density lipoprotein; HDL: high density lipoprotein

All results are adjusted for the following baseline (recruitment / 16 weeks) characteristics: Age, Parity, Ethnicity, BMI and clinical centre.

## Table S4: Mean concentration at 16-weeks of gestation and mean rate of change concentration per 4 weeks of gestational age between 16- and 36-weeks of gestation for each metabolic trait in obese pregnant women in the UPBEAT RCT (N = 115).

|  | Mean concentration at 16-weeks (95%CI)* | Mean change in concentration per 4 weeks gestational age (95%CI)* |
| --- | --- | --- |
| **Extremely large VLDL** | | |
| Concentration of chylomicrons and extremely large VLDL particles (mol/l) | 9.633x10^-11^  (6.954x10^-11^, 1.231x10^-10^ ) | 2.283x10^-11^  (2.072x10^-11^, 2.493x10^-11^) |
| Total lipids in chylomicrons and extremely large VLDL (mmol/l) | 0.021 (0.015, 0.027) | 0.005 (0.005, 0.005) |
| Phospholipids in chylomicrons and extremely large VLDL (mmol/l) | 0.003 (0.002, 0.003) | 7.630x10^-4^  (7.033x10^-4^ , 8.227x10^-4^ ) |
| Total cholesterol in chylomicrons and extremely large VLDL (mmol/l) | 0.004 (0.003, 0.005) | 0.001  (0.001, 0.001) |
| Cholesterol esters in chylomicrons and extremely large VLDL (mmol/l) | 0.002 (0.002, 0.003) | 6.248x10^-4^  (5.825x10^-4^ , 6.671x10^-4^ ) |
| Free cholesterol in chylomicrons and extremely large VLDL (mmol/l) | 0.002 (0.001, 0.002) | 5.268x10^-4^  (4.882x10^-4^ , 5.654x10^-4^ ) |
| Triglycerides in chylomicrons and extremely large VLDL (mmol/l) | 0.014 (0.010, 0.018) | 0.003 (0.003, 0.003) |
| **Very large VLDL** | | |
| Concentration of very large VLDL particles (mol/l) | 6.901x10^-10^  (5.279x10^-10^ , 8.524x10^-10^ ) | 1.702x10^-10^  (1.580x10^-10^ , 1.824x10^-10^ ) |
| Total lipids in very large VLDL (mmol/l) | 0.067 (0.051, 0.083) | 0.017 (0.015, 0.018) |
| Phospholipids in very large VLDL (mmol/l) | 0.011 (0.008, 0.013) | 0.003 (0.003, 0.003) |
| Total cholesterol in very large VLDL (mmol/l) | 0.013 (0.010, 0.016) | 0.003 (0.003, 0.004) |
| Cholesterol esters in very large VLDL (mmol/l) | 0.007 (0.006, 0.009) | 0.002 (0.002, 0.002) |
| Free cholesterol in very large VLDL (mmol/l) | 0.006 (0.004, 0.007) | 0.002 (0.001, 0.002) |
| Triglycerides in very large VLDL (mmol/l) | 0.043 (0.033, 0.053) | 0.010 (0.010, 0.011) |

**Table S4: continued**

|  | Mean concentration at 16-weeks (95%CI)* | Change in mean concentration per 4 weeks gestational age (95%CI)* |
| --- | --- | --- |
| **Large VLDL** | | |
| Concentration of large VLDL particles (mol/l) | 4.702x10^-9^  (3.788x10^-9^, 5.617x10^-9^) | 1.050x10^-9^  (9.812x10^-10^ , 1.119x10^-9^) |
| Total lipids in large VLDL (mmol/l) | 0.271 (0.218, 0.324) | 0.061 (0.057, 0.065) |
| Phospholipids in large VLDL (mmol/l) | 0.049 (0.039, 0.059) | 0.012 (0.011, 0.012) |
| Total cholesterol in large VLDL (mmol/l) | 0.060 (0.048, 0.072) | 0.014 (0.013, 0.015) |
| Cholesterol esters in large VLDL (mmol/l) | 0.032 (0.026, 0.038) | 0.006 (0.006, 0.007) |
| Free cholesterol in large VLDL (mmol/l) | 0.028 (0.021, 0.034) | 0.008 (0.007, 0.008) |
| Triglycerides in large VLDL (mmol/l) | 0.162 (0.131, 0.194) | 0.036 (0.033, 0.038) |
| **Medium VLDL** | | |
| Concentration of medium VLDL particles (mol/l) | 1.648x10^-8^  (1.415x10^-8^, 1.881x10^-8^) | 2.819x10^-9^  (2.647x10^-9^, 2.991x10^-9^) |
| Total lipids in medium VLDL (mmol/l) | 0.550 (0.472, 0.627) | 0.095 (0.089, 0.100) |
| Phospholipids in medium VLDL (mmol/l) | 0.110 (0.095, 0.125) | 0.019 (0.018, 0.020) |
| Total cholesterol in medium VLDL (mmol/l) | 0.149 (0.129, 0.169) | 0.025 (0.024, 0.027) |
| Cholesterol esters in medium VLDL (mmol/l) | 0.085 (0.074, 0.095) | 0.012 (0.012, 0.013) |
| Free cholesterol in medium VLDL (mmol/l) | 0.064 (0.054, 0.074) | 0.013 (0.012, 0.014) |
| Triglycerides in medium VLDL (mmol/l) | 0.291 (0.248, 0.335) | 0.050 (0.047, 0.054) |
| **Small VLDL** |  |  |
| Concentration of small VLDL particles (mol/l) | 2.838x10^-8^  (2.557x10^-8^, 3.120x10^-8^) | 3.813x10^-9^  (3.600x10^-9^, 4.026x10^-9^) |
| Total lipids in small VLDL (mmol/l) | 0.559 (0.505, 0.613) | 0.074 (0.070, 0.078) |
| Phospholipids in small VLDL (mmol/l) | 0.131 (0.120, 0.143) | 0.016 (0.015, 0.016) |
| Total cholesterol in small VLDL (mmol/l) | 0.205 (0.186, 0.224) | 0.025 (0.023, 0.026) |
| Cholesterol esters in small VLDL (mmol/l) | 0.124 (0.112, 0.137) | 0.013 (0.013, 0.014) |
| Free cholesterol in small VLDL (mmol/l) | 0.081 (0.073, 0.088) | 0.011 (0.010, 0.012) |
| Triglycerides in small VLDL (mmol/l) | 0.222 (0.196, 0.249) | 0.034 (0.032, 0.036) |

**Table S4: continued**

|  | Mean concentration at 16-weeks (95%CI)* | Change in mean concentration per 4 weeks gestational age (95%CI)* |
| --- | --- | --- |
| **Very small VLDL** | | |
| Concentration of very small VLDL particles (mol/l) | 3.608x10^-8^  (3.318x10^-8^, 3.897x10^-8^) | 3.735x10^-9^  (3.510x10^-9^, 3.960x10^-9^) |
| Total lipids in very small VLDL (mmol/l) | 0.456 (0.419, 0.493) | 0.045 (0.043, 0.048) |
| Phospholipids in very small VLDL (mmol/l) | 0.129 (0.117, 0.141) | 0.013 (0.012, 0.014) |
| Total cholesterol in very small VLDL (mmol/l) | 0.224 (0.207, 0.242) | 0.017 (0.016, 0.018) |
| Cholesterol esters in very small VLDL (mmol/l) | 0.153 (0.142, 0.165) | 0.010 (0.009, 0.011) |
| Free cholesterol in very small VLDL (mmol/l) | 0.071 (0.065, 0.077) | 0.007 (0.007, 0.008) |
| Triglycerides in very small VLDL (mmol/l) | 0.103 (0.092, 0.113) | 0.016 (0.015, 0.016) |
| **IDL** | | |
| Concentration of IDL particles (mol/l) | 9.478x10^-8^  (8.690x10^-8^, 1.027x10^-7^) | 7.630x10^-9^  (7.099x10^-9^, 8.161x10^-9^) |
| Total lipids in IDL (mmol/l) | 0.955 (0.874, 1.036) | 0.073 (0.068, 0.079) |
| Phospholipids in IDL (mmol/l) | 0.254 (0.234, 0.274) | 0.015 (0.014, 0.017) |
| Total cholesterol in IDL (mmol/l) | 0.594 (0.541, 0.647) | 0.041 (0.038, 0.045) |
| Cholesterol esters in IDL (mmol/l) | 0.426 (0.388, 0.463) | 0.031 (0.028, 0.033) |
| Free cholesterol in IDL (mmol/l) | 0.168 (0.152, 0.184) | 0.011 (0.010, 0.012) |
| Triglycerides in IDL (mmol/l) | 0.107 (0.097, 0.118) | 0.017 (0.016, 0.018) |
| **Large LDL** | | |
| Concentration of large LDL particles (mol/l) | 1.526x10^-7^  (1.390x10^-7^, 1.663x10^-7^) | 1.269x10^-8^  (1.178x10^-8^, 1.360x10^-8^) |
| Total lipids in large LDL (mmol/l) | 1.082 (0.985, 1.180) | 0.086 (0.080, 0.093) |
| Phospholipids in large LDL (mmol/l) | 0.279 (0.259, 0.299) | 0.016 (0.015, 0.017) |
| Total cholesterol in large LDL (mmol/l) | 0.707 (0.636, 0.778) | 0.056 (0.051, 0.060) |
| Cholesterol esters in large LDL (mmol/l) | 0.500 (0.447, 0.553) | 0.044 (0.040, 0.047) |
| Free cholesterol in large LDL (mmol/l) | 0.207 (0.189, 0.225) | 0.012 (0.011, 0.013) |
| Triglycerides in large LDL (mmol/l) | 0.096 (0.086, 0.105) | 0.014 (0.014, 0.015) |

**Table S4: continued**

|  | Mean concentration at 16-weeks (95%CI) | Change in mean concentration per 4 weeks gestational age (95%CI) |
| --- | --- | --- |
| **Medium LDL** | | |
| Concentration of medium LDL particles (mol/l) | 1.185x10^-7^  (1.069x10^-7^, 1.301x10^-7^) | 1.099x10^-8^  (1.021x10^-8^, 1.177x10^-8^) |
| Total lipids in medium LDL (mmol/l) | 0.602 (0.544, 0.661) | 0.054 (0.050, 0.058) |
| Phospholipids in medium LDL (mmol/l) | 0.171 (0.160, 0.183) | 0.011 (0.011, 0.012) |
| Total cholesterol in medium LDL (mmol/l) | 0.384 (0.339, 0.428) | 0.035 (0.033, 0.038) |
| Cholesterol esters in medium LDL (mmol/l) | 0.264 (0.227, 0.300) | 0.028 (0.026, 0.031) |
| Free cholesterol in medium LDL (mmol/l) | 0.120 (0.112, 0.128) | 0.007 (0.007, 0.008) |
| Triglycerides in medium LDL (mmol/l) | 0.047 (0.042, 0.051) | 0.007 (0.007, 0.007) |
| **Small LDL** | | |
| Concentration of small LDL particles (mol/l) | 1.405x10^-7^  (1.278x10^-7^, 1.531x10^-7^) | 1.207x10^-8^  (1.124x10^-8^, 1.289x10^-8^) |
| Total lipids in small LDL (mmol/l) | 0.393 (0.357, 0.428) | 0.033 (0.030, 0.035) |
| Phospholipids in small LDL (mmol/l) | 0.128 (0.121, 0.136) | 0.007 (0.007, 0.008) |
| Total cholesterol in small LDL (mmol/l) | 0.235 (0.208, 0.261) | 0.021 (0.019, 0.023) |
| Cholesterol esters in small LDL (mmol/l) | 0.163 (0.140, 0.185) | 0.016 (0.015, 0.018) |
| Free cholesterol in small LDL (mmol/l) | 0.072 (0.067, 0.077) | 0.004 (0.004, 0.005) |
| Triglycerides in small LDL (mmol/l) | 0.030 (0.027, 0.033) | 0.005 (0.004, 0.005) |
| **Very large HDL** | | |
| Concentration of very large HDL particles (mol/l) | 6.020x10^-7^  (5.372x10^-7^, 6.669x10^-7^) | 1.599x10^-8^  (1.341x10^-8^, 1.858x10^-8^) |
| Total lipids in very large HDL (mmol/l) | 0.608 (0.542, 0.674) | 0.016 (0.013, 0.018) |
| Phospholipids in very large HDL (mmol/l) | 0.309 (0.275, 0.344) | 0.005 (0.003, 0.006) |
| Total cholesterol in very large HDL (mmol/l) | 0.277 (0.248, 0.307) | 0.008 (0.006, 0.009) |
| Cholesterol esters in very large HDL (mmol/l) | 0.200 (0.179, 0.221) | 0.006 (0.005, 0.007) |
| Free cholesterol in very large HDL (mmol/l) | 0.077 (0.068, 0.086) | 0.002 (0.002, 0.002) |
| Triglycerides in very large HDL (mmol/l) | 0.021 (0.018, 0.024) | 0.003 (0.003, 0.003) |

**Table S4: continued**

|  | Mean concentration at 16-weeks (95%CI) | Change in mean concentration per 4 weeks gestational age (95%CI) |
| --- | --- | --- |
| **Large HDL** | | |
| Concentration of large HDL particles (mol/l) | 1.643x10^-6^  (1.497x10^-6^, 1.790x10^-6^) | 2.113x10^-9^  (-4.657x10^-9^, 8.883x10^-9^) |
| Total lipids in large HDL (mmol/l) | 1.034 (0.941, 1.128) | -4.406x10^-4^  (-0.005, 0.004) |
| Phospholipids in large HDL (mmol/l) | 0.498 (0.458, 0.538) | -0.002 (-0.004, 6.452x10^-5^) |
| Total cholesterol in large HDL (mmol/l) | 0.495 (0.445, 0.545) | -0.003 (-0.005, -6.039x10^-4^ ) |
| Cholesterol esters in large HDL (mmol/l) | 0.387 (0.349, 0.425) | -0.002 (-0.003, -1.133x10^-4^ ) |
| Free cholesterol in large HDL (mmol/l) | 0.108 (0.096, 0.120) | -0.001 (-0.002, -4.887x10^-4^ ) |
| Triglycerides in large HDL (mmol/l) | 0.040 (0.035, 0.045) | 0.004 (0.004, 0.005) |
| **Medium HDL** | | |
| Concentration of medium HDL particles (mol/l) | 2.283x10^-6^  (2.173x10^-6^, 2.393x10^-6^) | -3.467x10^-8^  (-4.120x10^-8^, -2.815x10^-8^) |
| Total lipids in medium HDL (mmol/l) | 0.968 (0.921, 1.016) | -0.017 (-0.019, -0.014) |
| Phospholipids in medium HDL (mmol/l) | 0.453 (0.432, 0.474) | -0.004 (-0.005, -0.002) |
| Total cholesterol in medium HDL (mmol/l) | 0.468 (0.441, 0.494) | -0.015 (-0.017, -0.014) |
| Cholesterol esters in medium HDL (mmol/l) | 0.377 (0.356, 0.398) | -0.014 (-0.015, -0.012) |
| Free cholesterol in medium HDL (mmol/l) | 0.090 (0.085, 0.096) | -0.002 (-0.002, -0.001) |
| Triglycerides in medium HDL (mmol/l) | 0.045 (0.042, 0.049) | 0.003 (0.002, 0.003) |

**Table S4: continued**

|  | Mean concentration at 16-weeks (95%CI)* | Change in mean concentration per 4 weeks gestational age (95%CI)* |
| --- | --- | --- |
| **Small HDL** | | |
| Concentration of small HDL particles (mol/l) | 5.042x10^-6^  (4.893x10^-6^, 5.191x10^-6^) | -2.350x10^-10^  (-8.239x10^-9^, 7.769x10^-9^) |
| Total lipids in small HDL (mmol/l) | 1.119 (1.086, 1.151) | -0.001 (-0.003, 6.969x10^-4^ ) |
| Phospholipids in small HDL (mmol/l) | 0.611 (0.586, 0.636) | -0.007 (-0.008, -0.005) |
| Total cholesterol in small HDL (mmol/l) | 0.452 (0.436, 0.467) | 7.082x10^-4^  (-3.675x10^-4^ , 0.002) |
| Cholesterol esters in small HDL (mmol/l) | 0.335 (0.320, 0.350) | 8.876x10^-4^  (-1.397x10^-4^ , 0.002) |
| Free cholesterol in small HDL (mmol/l) | 0.115 (0.111, 0.119) | -2.085x10^-4^  (-4.291x10^-4^ , 1.220x10^-5^) |
| Triglycerides in small HDL (mmol/l) | 0.048 (0.044, 0.052) | 0.005 (0.004, 0.005) |
| **Lipoprotein particle size** | | |
| Mean diameter for VLDL particles (nm) | 36.822 (36.484, 37.160) | 0.186 (0.169, 0.204) |
| Mean diameter for LDL particles (nm) | 23.599 (23.579, 23.619) | -0.004 (-0.005, -0.003) |
| Mean diameter for HDL particles | 10.158 (10.096, 10.221) | 0.004 (0.001, 0.006) |
| **Cholesterol** | | |
| Serum total cholesterol (mmol/l) | 4.222 (3.951, 4.492) | 0.229 (0.212, 0.246) |
| Total cholesterol in VLDL (mmol/l) | 0.657 (0.594, 0.720) | 0.085 (0.081, 0.090) |
| Remnant cholesterol (non-HDL, non-LDL -cholesterol) (mmol/l) | 1.252 (1.147, 1.357) | 0.126 (0.119, 0.134) |
| Total cholesterol in LDL (mmol/l) | 1.326 (1.184, 1.468) | 0.112 (0.103, 0.121) |
| Total cholesterol in HDL (mmol/l) | 1.691 (1.598, 1.783) | -0.010 (-0.015, -0.005) |
| Total cholesterol in HDL2 (mmol/l) | 1.160 (1.075, 1.246) | -0.019 (-0.023, -0.014) |
| Total cholesterol in HDL3 (mmol/l) | 0.527 (0.518, 0.537) | 0.009 (0.008, 0.009) |
| Esterified cholesterol (mmol/l) | 2.814 (2.607, 3.021) | 0.158 (0.145, 0.172) |
| Free cholesterol (mmol/l) | 1.308 (1.216, 1.400) | 0.069 (0.062, 0.076) |

**Table S4: continued**

|  | Mean concentration at 16-weeks (95%CI)* | Change in mean concentration per 4 weeks gestational age (95%CI)* |
| --- | --- | --- |
| **Glycerides and phospholipids** | | |
| Serum total triglycerides (mmol/l) | 1.273 (1.125, 1.421) | 0.206 (0.195, 0.218) |
| Triglycerides in VLDL (mmol/l) | 0.838 (0.716, 0.959) | 0.149 (0.140, 0.158) |
| Triglycerides in LDL (mmol/l) | 0.172 (0.155, 0.189) | 0.026 (0.024, 0.027) |
| Triglycerides in HDL (mmol/l) | 0.153 (0.141, 0.165) | 0.015 (0.014, 0.015) |
| Total phosphoglycerides (mmol/l) | 2.239 (2.133, 2.345) | 0.093 (0.085, 0.100) |
| Ratio of triglycerides to phosphoglycerides | 0.651 (0.597, 0.704) | 0.045 (0.042, 0.048) |
| Phosphatidylcholine and other cholines (mmol/l) | 2.052 (1.947, 2.157) | 0.097 (0.090, 0.104) |
| Sphingomyelins (mmol/l) | 0.396 (0.369, 0.422) | 0.008 (0.006, 0.010) |
| Total cholines (mmol/l) | 2.529 (2.410, 2.648) | 0.094 (0.085, 0.103) |
| **Apolipoproteins** | | |
| Apolipoprotein A-I (g/l) | 1.662 (1.606, 1.717) | 0.018 (0.015, 0.021) |
| Apolipoprotein B (g/l) | 0.794 (0.741, 0.846) | 0.067 (0.064, 0.071) |
| **Apolipoprotein Ratio** | | |
| Ratio of apolipoprotein B to apolipoprotein A-I | 0.493 (0.463, 0.522) | 0.032 (0.031, 0.034) |
| **Fatty acids** | | |
| Total fatty acids (mmol/l) | 12.627 (11.894, 13.359) | 0.777 (0.723, 0.831) |
| Estimated degree of unsaturation | 1.118 (1.105, 1.131) | -0.007 (-0.008, -0.007) |
| 22:6, docosahexaenoic acid (mmol/l) | 0.211 (0.196, 0.225) | 0.003 (0.002, 0.004) |
| 18:2, linoleic acid (mmol/l) | 3.462 (3.250, 3.673) | 0.182 (0.166, 0.197) |
| Omega-3 fatty acids (mmol/l) | 0.594 (0.549, 0.639) | 0.018 (0.015, 0.021) |
| Omega-6 fatty acids (mmol/l) | 3.913 (3.688, 4.138) | 0.166 (0.150, 0.183) |
| Polyunsaturated fatty acids (mmol/l) | 4.511 (4.251, 4.770) | 0.184 (0.166, 0.203) |
| Monounsaturated fatty acids; 16:1, 18:1 (mmol/l) | 3.558 (3.318, 3.798) | 0.294 (0.277, 0.311) |
| Saturated fatty acids (mmol/l) | 4.590 (4.313, 4.866) | 0.297 (0.276, 0.318) |

**Table S4: continued**

|  | Mean concentration at 16-weeks (95%CI)* | Change in mean concentration per 4 weeks gestational age (95%CI)* |
| --- | --- | --- |
| **Fatty acid ratios** | | |
| Ratio of 22:6 docosahexaenoic acid to total fatty acids (%) | 1.672 (1.595, 1.748) | -0.061 (-0.067, -0.056) |
| Ratio of 18:2 linoleic acid to total fatty acids (%) | 27.209 (26.460, 27.959) | -0.099 (-0.143, -0.056) |
| Ratio of omega-3 fatty acids to total fatty acids (%) | 4.663 (4.464, 4.863) | -0.113 (-0.123, -0.103) |
| Ratio of omega-6 fatty acids to total fatty acids (%) | 30.969 (30.261, 31.678) | -0.400 (-0.440, -0.360) |
| Ratio of polyunsaturated fatty acids to total fatty acids (%) | 35.670 (34.897, 36.443) | -0.513 (-0.556, -0.470) |
| Ratio of monounsaturated fatty acids to total fatty acids (%) | 27.690 (27.069, 28.310) | 0.436 (0.390, 0.482) |
| Ratio of saturated fatty acids to total fatty acids (%) | 36.555 (36.180, 36.931) | 0.045 (0.018, 0.072) |
| **Glycolysis related metabolites** | | |
| Glucose (mmol/l) | 3.764 (3.551, 3.977) | 0.074 (0.054, 0.093) |
| Lactate (mmol/l) | 1.061 (0.937, 1.185) | 0.039 (0.029, 0.049) |
| Pyruvate (mmol/l) | 0.088 (0.077, 0.099) | 0.005 (0.004, 0.006) |
| Citrate (mmol/l) | 0.109 (0.104, 0.114) | 0.004 (0.003, 0.004) |
| Glycerol (mmol/l) | 0.064 (0.058, 0.070) | -0.001 (-0.001, -5.240x10^-4^ ) |
| **Amino acids** |  |  |
| Alanine (mmol/l) | 0.378 (0.366, 0.390) | 0.007 (0.006, 0.007) |
| Glutamine (mmol/l) | 0.407 (0.395, 0.420) | -6.445x10^-4^  (-0.002, 2.203x10^-4^ ) |
| Glycine (mmol/l) | 0.206 (0.198, 0.214) | 0.002 (0.001, 0.002) |
| Histidine (mmol/l) | 0.064 (0.062, 0.066) | 2.780x10^-4^  (1.281x10^-4^ , 4.278x10^-4^ ) |
| **Amino acids - branched chain** | | |
| Isoleucine (mmol/l) | 0.047 (0.043, 0.050) | 7.648x10^-4^  (4.956x10^-4^ , 0.001) |
| Leucine (mmol/l) | 0.062 (0.058, 0.065) | 2.006x10^-5^  (-2.551x10^-4^ , 2.952x10^-4^ ) |
| Valine (mmol/l) | 0.131 (0.123, 0.138) | -0.004 (-0.005, -0.004) |

**Table S4: continued**

|  | Mean concentration at 16-weeks (95%CI)* | Change in mean concentration per 4 weeks gestational age (95%CI)* |
| --- | --- | --- |
| **Amino acids - aromatic** | | |
| Phenylalanine (mmol/l) | 0.072 (0.069, 0.075) | 0.001 (9.617x10^-4^ , 0.001) |
| Tyrosine (mmol/l) | 0.038 (0.036, 0.040) | -2.555x10^-4^  (-4.270x10^-4^ , -8.403x10^-5^) |
| **Ketone bodies** | | |
| Acetate (mmol/l) | 0.041 (0.039, 0.044) | 1.444x10^-4^  (-5.902x10^-5^, 3.477x10^-4^ ) |
| Acetoacetate (mmol/l) | 0.021 (0.018, 0.024) | 3.697x10^-4^  (9.760x10^-5^, 6.419x10^-4^ ) |
| 3-hydroxybutyrate (mmol/l) | 0.078 (0.062, 0.093) | 0.005 (0.003, 0.006) |
| **Fluid balance** | | |
| Creatinine (mmol/l) | 0.036 (0.034, 0.038) | 2.231x10^-4^  (9.232x10^-5^, 3.539x10^-4^ ) |
| Albumin (mmol/l) | 0.084 (0.083, 0.085) | -5.740x10^-4^  (-6.492x10^-4^ , -4.988x10^-4^ ) |
| **Inflammation** | | |
| Glycoprotein acetyls, mainly a1-acid glycoprotein (mmol/l) | 1.540 (1.495, 1.585) | 0.047 (0.044, 0.050) |

* Units for each metabolic measure are provided in column one with the metabolic measure name. The results for mean levels are 16-weeks of gestation are these units; results for the change between 16 and 36 weeks are these units per 4 week of gestation.

VLDL: very low density lipoprotein; LDL: low density lipoprotein; IDL: intermediate density lipoprotein; HDL: high density lipoprotein

All results are adjusted for the following baseline (recruitment / 16 weeks) characteristics: Age, Parity, Ethnicity, BMI and clinical centre.

## Table S5: Effect of the UPBEAT diet and physical activity lifestyle intervention on metabolic profiles: difference in mean rate of change in metabolic traits (original units) comparing women receiving intervention to the control group. N = 1158

|  | Difference in mean rate of change in traits per 4 weeks of gestation between 16 and 36 weeks between women receiving intervention and control group (reference) | p-value |
| --- | --- | --- |
| **Extremely large VLDL** | | |
| Concentration of chylomicrons and extremely large VLDL particles (mol/l) | -5.430x10^-12^ (-8.323x10^-12^, -2.537x10^-12^) | 2.48x10^-4^ |
| Total lipids in chylomicrons and extremely large VLDL (mmol/l) | -0.001 (-0.002, -5.322x10^-4^ ) | 2.87x10^-4^ |
| Phospholipids in chylomicrons and extremely large VLDL (mmol/l) | -1.457x10^-4^  (-2.287x10^-4^ , -6.274x10^-5^) | 6.06x10^-4^ |
| Total cholesterol in chylomicrons and extremely large VLDL (mmol/l) | -1.623x10^-4^  (-2.725x10^-4^ , -5.212x10^-5^) | 0.004 |
| Cholesterol esters in chylomicrons and extremely large VLDL (mmol/l) | -6.952x10^-5^ (-1.289x10^-4^ , -1.015x10^-5^) | 0.022 |
| Free cholesterol in chylomicrons and extremely large VLDL (mmol/l) | -9.276x10^-5^ (-1.468x10^-4^ , -3.868x10^-5^) | 8.08x10^-4^ |
| Triglycerides in chylomicrons and extremely large VLDL (mmol/l) | -8.450x10^-4^  (-0.001, -4.077x10^-4^ ) | 1.62x10^-4^ |
| **Very large VLDL** | | |
| Concentration of very large VLDL particles (mol/l) | -2.919x10^-11^ (-4.656x10^-11^, -1.183x10^-11^) | 0.001 |
| Total lipids in very large VLDL (mmol/l) | -0.003 (-0.005, -0.001) | 0.001 |
| Phospholipids in very large VLDL (mmol/l) | -4.618x10^-4^  (-7.453x10^-4^ , -1.782x10^-4^ ) | 0.001 |
| Total cholesterol in very large VLDL (mmol/l) | -4.869x10^-4^  (-8.058x10^-4^ , -1.681x10^-4^ ) | 0.003 |
| Cholesterol esters in very large VLDL (mmol/l) | -2.409x10^-4^  (-4.085x10^-4^ , -7.328x10^-5^) | 0.005 |
| Free cholesterol in very large VLDL (mmol/l) | -2.459x10^-4^  (-3.986x10^-4^ , -9.316x10^-5^) | 0.002 |
| Triglycerides in very large VLDL (mmol/l) | -0.002 (-0.003, -7.781x10^-4^ ) | 8.16x10^-4^ |
| **Large VLDL** | | |
| Concentration of large VLDL particles (mol/l) | -1.436x10^-10^ (-2.419x10^-10^ , -4.524x10^-11^) | 0.004 |
| Total lipids in large VLDL (mmol/l) | -0.008 (-0.014, -0.003) | 0.005 |
| Phospholipids in large VLDL (mmol/l) | -0.001 (-0.003, -4.202x10^-4^ ) | 0.006 |
| Total cholesterol in large VLDL (mmol/l) | -0.002 (-0.003, -3.658x10^-4^ ) | 0.011 |
| Cholesterol esters in large VLDL (mmol/l) | -6.322x10^-4^  (-0.001, -4.684x10^-5^) | 0.035 |
| Free cholesterol in large VLDL (mmol/l) | -9.788x10^-4^  (-0.002, -3.053x10^-4^ ) | 0.004 |
| Triglycerides in large VLDL (mmol/l) | -0.005 (-0.009, -0.002) | 0.003 |

**Table S5: continued**

| **Medium VLDL** | | | |
| --- | --- | --- | --- |
| Concentration of medium VLDL particles (mol/l) | | -2.706x10^-10^ (-5.176x10^-10^ , -2.363x10^-11^) | 0.032 |
| Total lipids in medium VLDL (mmol/l) | | -0.009 (-0.017, -5.203x10^-4^ ) | 0.037 |
| Phospholipids in medium VLDL (mmol/l) | | -0.002 (-0.003, 2.571x10^-5^) | 0.054 |
| Total cholesterol in medium VLDL (mmol/l) | | -0.001 (-0.004, 6.072x10^-4^ ) | 0.165 |
| Cholesterol esters in medium VLDL (mmol/l) | | -3.934x10^-4^  (-0.001, 6.747x10^-4^ ) | 0.471 |
| Free cholesterol in medium VLDL (mmol/l) | | -0.001 (-0.002, -5.763x10^-6^) | 0.049 |
| Triglycerides in medium VLDL (mmol/l) | | -0.006 (-0.010, -0.001) | 0.016 |
| **Small VLDL** | | | |
| Concentration of small VLDL particles (mmol/l) | | -8.340x10^-11^ (-3.894x10^-10^ , 2.226x10^-10^ ) | 0.593 |
| Total lipids in small VLDL (mmol/l) | | -0.001 (-0.007, 0.005) | 0.702 |
| Phospholipids in small VLDL (mmol/l) | | -2.123x10^-4^  (-0.001, 0.001) | 0.738 |
| Total cholesterol in small VLDL (mmol/l) | | 8.711x10^-4^  (-0.001, 0.003) | 0.421 |
| Cholesterol esters in small VLDL (mmol/l) | | 9.298x10^-4^  (-4.024x10^-4^ , 0.002) | 0.172 |
| Free cholesterol in small VLDL (mmol/l) | | -5.333x10^-5^ (-9.154x10^-4^ , 8.087x10^-4^ ) | 0.904 |
| Triglycerides in small VLDL (mmol/l) | | -0.002 (-0.005, 0.001) | 0.215 |
| **Very small VLDL** | | | |
| Concentration of very small VLDL particles (mmol/l) | 2.266x10^-10^ (-9.757x10^-11^, 5.508x10^-10^ ) | | 0.171 |
| Total lipids in very small VLDL (mmol/l) | 0.003 (-9.195x10^-4^ , 0.007) | | 0.13 |
| Phospholipids in very small VLDL (mmol/l) | 0.001 (-3.358x10^-5^, 0.003) | | 0.057 |
| Total cholesterol in very small VLDL (mmol/l) | 0.002 (-2.896x10^-5^, 0.004) | | 0.054 |
| Cholesterol esters in very small VLDL (mmol/l) | 0.001 (-7.809x10^-5^, 0.002) | | 0.067 |
| Free cholesterol in very small VLDL (mmol/l) | 7.239x10^-4^  (1.017x10^-5^, 0.001) | | 0.047 |
| Triglycerides in very small VLDL (mmol/l) | 6.178x10^-5^ (-0.001, 0.001) | | 0.922 |

**Table S5: continued**

| **IDL** | | |
| --- | --- | --- |
| Concentration of IDL particles (mmol/l) | 5.615x10^-10^ (-2.024x10^-10^ , 1.325x10^-9^) | 0.15 |
| Total lipids in IDL (mmol/l) | 0.006 (-0.002, 0.014) | 0.132 |
| Phospholipids in IDL (mmol/l) | 0.002 (-1.812x10^-4^ , 0.004) | 0.077 |
| Total cholesterol in IDL (mmol/l) | 0.004 (-0.001, 0.009) | 0.133 |
| Cholesterol esters in IDL (mmol/l) | 0.002 (-0.001, 0.006) | 0.216 |
| Free cholesterol in IDL (mmol/l) | 0.002 (9.352x10^-5^, 0.003) | 0.037 |
| Triglycerides in IDL (mmol/l) | 4.641x10^-4^  (-8.328x10^-4^ , 0.002) | 0.483 |
| **Large LDL** |  |  |
| Concentration of large LDL particles (mol/l) | 9.960x10^-10^ (-3.150x10^-10^ , 2.307x10^-9^) | 0.137 |
| Total lipids in large LDL (mmol/l) | 0.007 (-0.002, 0.017) | 0.121 |
| Phospholipids in large LDL (mmol/l) | 0.001 (-4.370x10^-4^ , 0.003) | 0.135 |
| Total cholesterol in large LDL (mmol/l) | 0.006 (-0.001, 0.012) | 0.098 |
| Cholesterol esters in large LDL (mmol/l) | 0.004 (-0.001, 0.009) | 0.121 |
| Free cholesterol in large LDL (mmol/l) | 0.002 (-1.379x10^-5^, 0.003) | 0.052 |
| Triglycerides in large LDL (mmol/l) | 3.603x10^-4^  (-7.183x10^-4^ , 0.001) | 0.513 |
| **Medium LDL** | | |
| Concentration of medium LDL particles (mol/l) | 8.685x10^-10^ (-2.488x10^-10^ , 1.986x10^-9^) | 0.128 |
| Total lipids in medium LDL (mmol/l) | 0.004 (-0.001, 0.010) | 0.121 |
| Phospholipids in medium LDL (mmol/l) | 4.492x10^-4^  (-5.950x10^-4^ , 0.001) | 0.399 |
| Total cholesterol in medium LDL (mmol/l) | 0.004 (-3.480x10^-4^ , 0.008) | 0.073 |
| Cholesterol esters in medium LDL (mmol/l) | 0.003 (-1.232x10^-4^ , 0.007) | 0.059 |
| Free cholesterol in medium LDL (mmol/l) | 5.072x10^-4^  (-2.358x10^-4^ , 0.001) | 0.181 |
| Triglycerides in medium LDL (mmol/l) | 1.647x10^-4^  (-3.576x10^-4^ , 6.870x10^-4^ ) | 0.537 |
| **Small LDL** | | |
| Concentration of small LDL particles (mol/l) | 8.393x10^-10^ (-3.470x10^-10^ , 2.025x10^-9^) | 0.166 |
| Total lipids in small LDL (mmol/l) | 0.002 (-8.675x10^-4^ , 0.006) | 0.149 |
| Phospholipids in small LDL (mmol/l) | 1.340x10^-4^  (-5.082x10^-4^ , 7.762x10^-4^ ) | 0.683 |
| Total cholesterol in small LDL (mmol/l) | 0.002 (-1.279x10^-4^ , 0.005) | 0.063 |
| Cholesterol esters in small LDL (mmol/l) | 0.002 (3.884x10^-5^, 0.004) | 0.046 |
| Free cholesterol in small LDL (mmol/l) | 2.574x10^-4^  (-1.803x10^-4^ , 6.951x10^-4^ ) | 0.249 |
| Triglycerides in small LDL (mmol/l) | -5.176x10^-5^ (-3.683x10^-4^ , 2.648x10^-4^ ) | 0.749 |

**Table S5: continued**

| **Very large HDL** | | |
| --- | --- | --- |
| Concentration of very large HDL particles (mol/l) | -1.228x10^-9^ (-4.887x10^-9^, 2.432x10^-9^) | 0.511 |
| Total lipids in very large HDL (mmol/l) | -0.001 (-0.005, 0.002) | 0.491 |
| Phospholipids in very large HDL (mmol/l) | 5.000x10^-5^ (-0.002, 0.002) | 0.96 |
| Total cholesterol in very large HDL (mmol/l) | -0.001 (-0.003, 6.460x10^-4^ ) | 0.215 |
| Cholesterol esters in very large HDL (mmol/l) | -8.292x10^-4^  (-0.002, 4.028x10^-4^ ) | 0.187 |
| Free cholesterol in very large HDL (mmol/l) | -2.818x10^-4^  (-8.186x10^-4^ , 2.550x10^-4^ ) | 0.304 |
| Triglycerides in very large HDL (mmol/l) | -2.479x10^-4^  (-4.714x10^-4^ , -2.436x10^-5^) | 0.03 |
| **Large HDL** | | |
| Concentration of large HDL particles (mol/l) | -1.478x10^-9^ (-1.098x10^-8^, 8.028x10^-9^) | 0.761 |
| Total lipids in large HDL  (mmol/l) | -8.993x10^-4^  (-0.007, 0.005) | 0.771 |
| Phospholipids in large HDL  (mmol/l) | -1.159x10^-4^  (-0.003, 0.003) | 0.934 |
| Total cholesterol in large HDL  (mmol/l) | -6.220x10^-4^  (-0.004, 0.002) | 0.695 |
| Cholesterol esters in large HDL  (mmol/l) | -5.784x10^-4^  (-0.003, 0.002) | 0.634 |
| Free cholesterol in large HDL  (mmol/l) | -4.172x10^-5^ (-7.685x10^-4^ , 6.851x10^-4^ ) | 0.91 |
| Triglycerides in large HDL  (mmol/l) | -1.699x10^-4^  (-5.560x10^-4^ , 2.162x10^-4^ ) | 0.389 |
| **Medium HDL** | | |
| Concentration of medium HDL particles (mol/l) | -3.573x10^-9^ (-1.261x10^-8^, 5.468x10^-9^) | 0.439 |
| Total lipids in medium HDL (mmol/l) | -0.001 (-0.005, 0.003) | 0.481 |
| Phospholipids in medium HDL (mmol/l) | -7.396x10^-4^  (-0.002, 9.798x10^-4^ ) | 0.399 |
| Total cholesterol in medium HDL (mmol/l) | -5.119x10^-4^  (-0.003, 0.002) | 0.657 |
| Cholesterol esters in medium HDL (mmol/l) | -4.907x10^-4^  (-0.002, 0.001) | 0.598 |
| Free cholesterol in medium HDL (mmol/l) | -2.018x10^-5^ (-4.787x10^-4^ , 4.383x10^-4^ ) | 0.931 |
| Triglycerides in medium HDL (mmol/l) | -1.433x10^-4^  (-4.170x10^-4^ , 1.305x10^-4^ ) | 0.305 |

**Table S5: continued**

| **Small HDL** | | |
| --- | --- | --- |
| Concentration of small HDL particles (mol/l) | -3.956x10^-9^ (-1.489x10^-8^, 6.978x10^-9^) | 0.478 |
| Total lipids in small HDL (mmol/l) | -6.961x10^-4^  (-0.003, 0.002) | 0.578 |
| Phospholipids in small HDL (mmol/l) | -0.002 (-0.004, -1.363x10^-4^ ) | 0.035 |
| Total cholesterol in small HDL (mmol/l) | 0.002 (1.270x10^-4^ , 0.003) | 0.033 |
| Cholesterol esters in small HDL (mmol/l) | 0.002 (5.153x10^-4^ , 0.003) | 0.008 |
| Free cholesterol in small HDL (mmol/l) | -3.001x10^-4^  (-6.091x10^-4^ , 8.987x10^-6^) | 0.057 |
| Triglycerides in small HDL (mmol/l) | -2.744x10^-4^  (-6.414x10^-4^ , 9.250x10^-5^) | 0.143 |
| **Lipoprotein particle size** | | |
| Mean diameter for VLDL particles (nm) | -0.031 (-0.054, -0.008) | 0.008 |
| Mean diameter for LDL particles (nm) | 1.061x10^-4^  (-0.001, 0.001) | 0.873 |
| Mean diameter for HDL particles (nm) | -3.594x10^-5^ (-0.004, 0.003) | 0.984 |
| **Cholesterol** | | |
| Serum total cholesterol (mol/l) | 0.014 (-0.010, 0.039) | 0.245 |
| Total cholesterol in VLDL (mmol/l) | -0.001 (-0.008, 0.006) | 0.76 |
| Remnant cholesterol (non-HDL, non-LDL -cholesterol) (mmol/l) | 0.003 (-0.008, 0.013) | 0.621 |
| Total cholesterol in LDL (mmol/l) | 0.012 (-0.001, 0.025) | 0.082 |
| Total cholesterol in HDL (mmol/l) | -4.998x10^-4^  (-0.007, 0.006) | 0.882 |
| Total cholesterol in HDL2 (mmol/l) | -3.999x10^-4^  (-0.007, 0.006) | 0.899 |
| Total cholesterol in HDL3 (mmol/l) | -8.860x10^-5^ (-8.504x10^-4^ , 6.732x10^-4^ ) | 0.82 |
| Esterified cholesterol (mmol/l) | 0.008 (-0.011, 0.028) | 0.387 |
| Free cholesterol (mmol/l) | 0.003 (-0.006, 0.013) | 0.466 |
| **Glycerides and phospholipids** | | |
| Serum total triglycerides (mol/l) | -0.015 (-0.031, 8.645x10^-4^ ) | 0.064 |
| Triglycerides in VLDL (mmol/l) | -0.015 (-0.028, -0.002) | 0.022 |
| Triglycerides in LDL (mmol/l) | 4.618x10^-4^  (-0.001, 0.002) | 0.635 |
| Triglycerides in HDL (mmol/l) | -8.483x10^-4^  (-0.002, 2.444x10^-4^ ) | 0.128 |
| Total phosphoglycerides (mmol/l) | -0.003 (-0.013, 0.007) | 0.569 |
| Ratio of triglycerides to phosphoglycerides | -0.005 (-0.009, -6.198x10^-4^ ) | 0.024 |
| Phosphatidylcholine and other cholines (mmol/l) | -0.001 (-0.011, 0.009) | 0.809 |
| Sphingomyelins (mmol/l) | 7.135x10^-4^  (-0.002, 0.003) | 0.586 |
| Total cholines (mmol/l) | -9.504x10^-4^  (-0.013, 0.011) | 0.875 |
| **Apolipoproteins** | | |
| Apolipoprotein A-I (g/l) | -0.001 (-0.005, 0.003) | 0.558 |
| Apolipoprotein B (g/l) | 6.907x10^-4^  (-0.005, 0.006) | 0.8 |
| **Apolipoproteins ratio** | | |
| Ratio of apolipoprotein B to apolipoprotein A-I | 7.824x10^-4^  (-0.002, 0.004) | 0.582 |

**Table S5: continued**

| **Fatty acids** | | |
| --- | --- | --- |
| Total fatty acids (mmol/l) | -0.036 (-0.109, 0.038) | 0.341 |
| Estimated degree of unsaturation | 0.001 (1.229x10^-4^ , 0.002) | 0.028 |
| 22:6, docosahexaenoic acid (mmol/l) | 3.139x10^-4^  (-9.443x10^-4^ , 0.002) | 0.625 |
| 18:2, linoleic acid (mmol/l) | 0.004 (-0.017, 0.025) | 0.716 |
| Omega-3 fatty acids (mmol/l) | -7.868x10^-4^  (-0.004, 0.003) | 0.669 |
| Omega-6 fatty acids (mmol/l) | 0.004 (-0.019, 0.026) | 0.735 |
| Polyunsaturated fatty acids (mmol/l) | 0.003 (-0.023, 0.029) | 0.816 |
| Monounsaturated fatty acids; 16:1, 18:1 (mmol/l) | -0.016 (-0.039, 0.008) | 0.191 |
| Saturated fatty acids | -0.021 (-0.050, 0.007) | 0.14 |
| **Fatty acid ratios** | | |
| Ratio of 22:6 docosahexaenoic acid to total fatty acids (%) | 0.005 (-0.001, 0.010) | 0.111 |
| Ratio of 18:2 linoleic acid to total fatty acids (%) | 0.069 (0.013, 0.126) | 0.017 |
| Ratio of omega-3 fatty acids to total fatty acids (%) | 0.003 (-0.010, 0.016) | 0.623 |
| Ratio of omega-6 fatty acids to total fatty acids (%) | 0.077 (0.023, 0.131) | 0.006 |
| Ratio of polyunsaturated fatty acids to total fatty acids (%) | 0.079 (0.020, 0.138) | 0.008 |
| Ratio of monounsaturated fatty acids to total fatty acids (%) | -0.025 (-0.073, 0.023) | 0.311 |
| Ratio of saturated fatty acids to total fatty acids (%) | -0.049 (-0.083, -0.015) | 0.005 |
| **Glycolysis related metabolites** | | |
| Glucose (mmol/l) | -0.013 (-0.037, 0.010) | 0.257 |
| Lactate (mmol/l) | -0.017 (-0.027, -0.006) | 0.003 |
| Pyruvate (mmol/l) | -0.002 (-0.003, -6.028x10^-4^ ) | 0.003 |
| Citrate (mmol/l) | -1.962x10^-4^  (-6.819x10^-4^ , 2.896x10^-4^ ) | 0.429 |
| Glycerol (mmol/l) | -9.708x10^-5^ (-6.139x10^-4^ , 4.197x10^-4^ ) | 0.713 |
| **Amino acids** | | |
| Alanine (mmol/l) | -0.001 (-0.002, -2.708x10^-5^) | 0.045 |
| Glutamine (mmol/l) | 3.303x10^-4^  (-8.107x10^-4^ , 0.001) | 0.571 |
| Glycine (mmol/l) | -2.205x10^-4^  (-8.150x10^-4^ , 3.741x10^-4^ ) | 0.468 |
| Histidine (mmol/l) | 3.324x10^-6^ (-1.746x10^-4^ , 1.813x10^-4^ ) | 0.971 |
| **Amino acids - branched chain** | | |
| Isoleucine (mmol/l) | -2.696x10^-4^  (-5.843x10^-4^ , 4.512x10^-5^) | 0.093 |
| Leucine (mmol/l) | -7.271x10^-5^ (-3.835x10^-4^ , 2.381x10^-4^ ) | 0.647 |
| Valine (mmol/l) | 1.108x10^-4^  (-4.927x10^-4^ , 7.143x10^-4^ ) | 0.719 |
| **Amino acids - aromatic** | | |
| Phenylalanine (mmol/l) | 5.374x10^-5^ (-2.019x10^-4^ , 3.094x10^-4^ ) | 0.68 |
| Tyrosine (mmol/l) | 1.131x10^-4^  (-7.143x10^-5^, 2.977x10^-4^ ) | 0.23 |

**Table S5: continued**

| **Ketone bodies** | | |
| --- | --- | --- |
| Acetate (mmol/l) | 3.081x10^-4^  (7.541x10^-5^, 5.408x10^-4^ ) | 0.01 |
| Acetoacetate (mmol/l) | -1.508x10^-4^  (-4.641x10^-4^ , 1.625x10^-4^ ) | 0.346 |
| 3-hydroxybutyrate (mmol/l) | 0.001 (-1.717x10^-4^ , 0.003) | 0.084 |
| **Fluid balance** | | |
| Creatinine (mmol/l) | 3.527x10^-5^ (-1.362x10^-4^ , 2.068x10^-4^ ) | 0.687 |
| Albumin (mmol/l) | -3.305x10^-5^ (-1.217x10^-4^ , 5.560x10^-5^) | 0.465 |
| **Inflammation** | | |
| Glycoprotein acetyls, mainly a1-acid glycoprotein (mmo/l) | -0.003 (-0.007, 6.824x10^-4^ ) | 0.105 |

Results are the difference in mean rate change of each trait in original units (see first column) per 4 weeks of gestation between 16 and 36 weeks.

VLDL: very low density lipoprotein; LDL: low density lipoprotein; IDL: intermediate density lipoprotein; HDL: high density lipoprotein

All results are adjusted for the following baseline (recruitment / 16 weeks) characteristics: Age, Parity, Ethnicity, BMI and clinical centre.

**Table S6: Correlations between estimates of mean slope from different sensitivity analyses.**

|  | **Main Analyses** | **MLM SD** | **MLM SD no outliers** | **Paired-t SD** | **Paired-t SD no outliers** | **MLM IQR** | **MLM IQR no outliers** | **Paired-t IQR** | **Paired-t IQR no outliers** | **Paired-t MAD** | **Paired-t MAD no outliers** |
| --- | --- | --- | --- | --- | --- | --- | --- | --- | --- | --- | --- |
| **Main Analyses** | 1 |  |  |  |  |  |  |  |  |  |  |
| **MLM SD** | 0.97 | 1 |  |  |  |  |  |  |  |  |  |
| **MLM SD no outliers** | 0.97 | 0.99 | 1 |  |  |  |  |  |  |  |  |
| **Paired-t SD** | 0.95 | 0.97 | 0.97 | 1 |  |  |  |  |  |  |  |
| **Paired-t SD no outliers** | 0.93 | 0.97 | 0.97 | 0.98 | 1 |  |  |  |  |  |  |
| **MLM IQR** | 0.97 | 1.00 | 0.99 | 0.97 | 0.96 | 1 |  |  |  |  |  |
| **MLM IQR no outliers** | 0.96 | 0.99 | 1.00 | 0.98 | 0.97 | 0.99 | 1 |  |  |  |  |
| **Paired-t IQR** | 0.94 | 0.95 | 0.96 | 0.99 | 0.98 | 0.97 | 0.96 | 1 |  |  |  |
| **Paired-t IQR no outliers** | 0.93 | 0.95 | 0.97 | 0.98 | 1.00 | 0.96 | 0.97 | 0.98 | 1 |  |  |
| **Paired-t MAD** | 0.93 | 0.95 | 0.95 | 0.99 | 0.97 | 0.96 | 0.96 | 1.00 | 0.98 | 1 |  |
| **Paired-t MAD no outliers** | 0.90 | 0.90 | 0.92 | 0.93 | 0.94 | 0.90 | 0.92 | 0.93 | 0.94 | 0.93 | 1 |

**Supplementary Figures**

**Figure S1: Stages and methods used for NMR platform metabolic measures (adapted from Wurtz et al.[**[**13**](#_ENREF_13)**])**

**Figure S2: Illustration of the timing of metabolite measurements.** This illustrates the wide gaps in time (gestational age) between measurements and therefore the inability to use smoothing/NON-linear models as we do not have any data between the three data collection points. For this reason we were only able to use linear spline models.

**
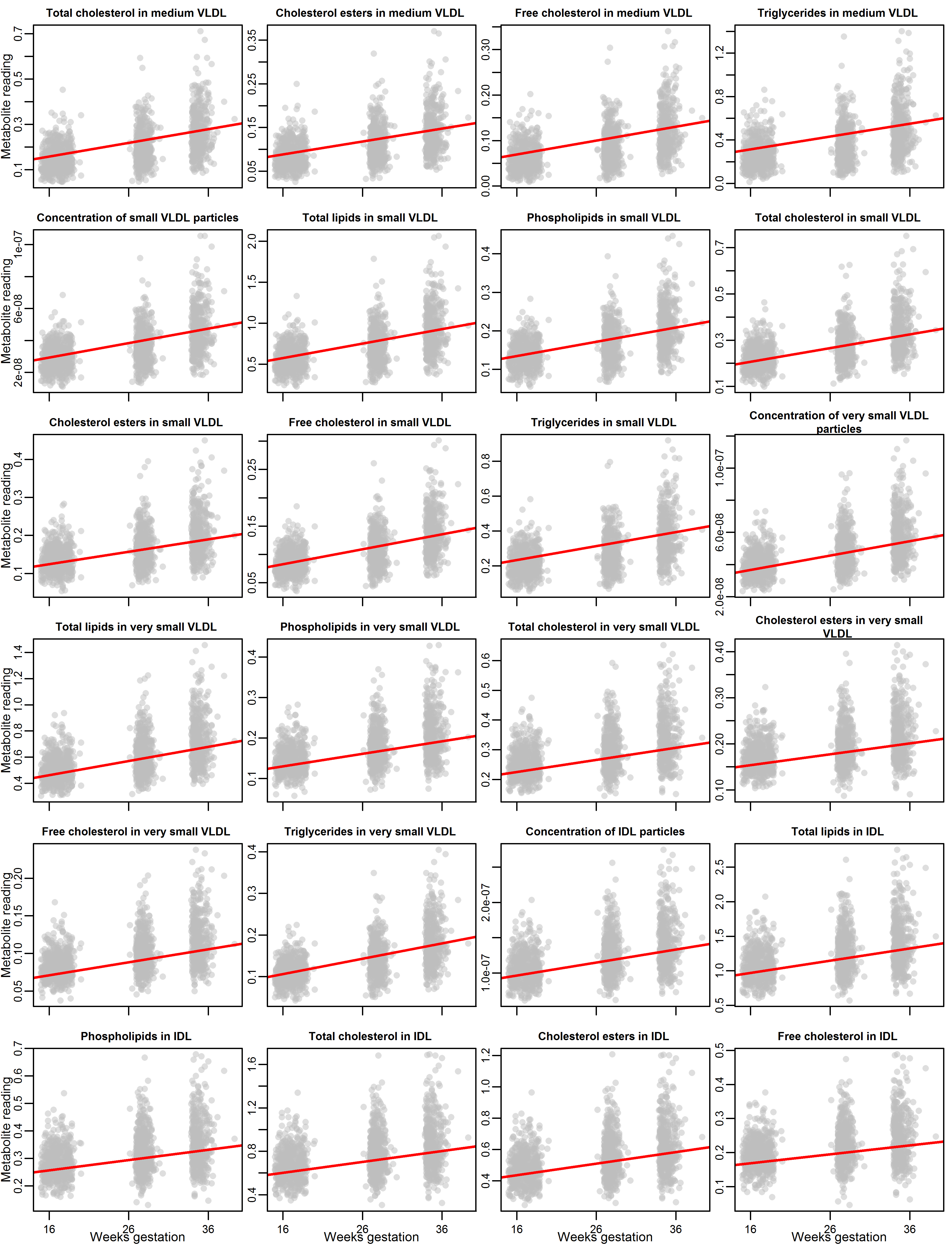
**

**Figure S3: Comparison of the effect of the UPBEAT intervention between 16 and 28 weeks of gestation to that between 28 and 36 weeks of gestation. N = 1158.**

Results in red are between 16 and 28 weeks and in blue between 28 and 36 weeks.

**Figure S3: Continued**

**Figure S3: Continued**

**Footnote to Figure S3**

All results are adjusted for the following baseline (recruitment / 16 weeks) characteristics: Age, Parity, Ethnicity, BMI and clinical centre.

**Figure S4: Comparison of results from our main multilevel model analyses and sensitivity analyses using generalised estimating equations for the effect of the UPBEAT intervention on change in metabolites**

**sReferences**

1. Soininen P, Kangas AJ, Wurtz P, Suna T, Ala-Korpela M: **Quantitative serum nuclear magnetic resonance metabolomics in cardiovascular epidemiology and genetics**. *Circulation Cardiovascular genetics* 2015, **8**(1):192-206.

2. Soininen P, Kangas AJ, Wurtz P, Tukiainen T, Tynkkynen T, Laatikainen R, Jarvelin MR, Kahonen M, Lehtimaki T, Viikari J *et al*: **High-throughput serum NMR metabonomics for cost-effective holistic studies on systemic metabolism**. *The Analyst* 2009, **134**(9):1781-1785.

3. Howe LD, Tilling K, Matijasevich A, Petherick ES, Santos AC, Fairley L, Wright J, Santos IS, Barros AJ, Martin RM *et al*: **Linear spline multilevel models for summarising childhood growth trajectories: A guide to their application using examples from five birth cohorts**. *Stat Methods Med Res* 2016, **25**:1854-1874.

4. Tilling K, Macdonald-Wallis C, Lawlor DA, Hughes RA, Howe LD: **Modelling childhood growth using fractional polynomials and linear splines**. *Ann Nutr Metab* 2014, **65**(2-3):129-138.

5. Poston L, Bell R, Croker H, Flynn AC, Godfrey KM, Goff L, Hayes L, Khazaezadeh N, Nelson SM, Oteng-Ntim E *et al*: **Effect of a behavioural intervention in obese pregnant women (the UPBEAT study): a multicentre, randomised controlled trial**. *The lancet Diabetes & endocrinology* 2015, **3**(10):767-777.

6. Wasserstein RL, Lazar NA: **The ASA's statement on p-values: context, process, and purpose**. *The American Statistician* 2016, **DOI: 10.1080/00031305.2016.1154108**.

7. Sterne JAC, Davey Smith G: **Shifting the evidence - what's wrong with significance tests?** *BMJ* 2001, **322**:226-231.

8. Altman D, Machin D, Bryant T, Hanley J: **Statistics with confidence. Second edition ed.**, Second edn. London: BMJ Books; 2000.

9. Benjamini J: **Discoverying the false discovery rate**. *Journal of the Royal Statistical Scociety, Series B* 2010, **72**:405-416.

10. Warrington NM, Tilling K, Howe LD, Paternoster L, Pennell CE, Wu YY, Briollais L: **Robustness of the linear mixed effects model to error distribution assumptions and the consequences for genome-wide association studies**. *Stat Appl Genet Mol Biol* 2014, **13**(5):567-587.

11. Wilcox RR, Rousselet GA: **A guide to robust statistical methods in neuroscience** *bioRxiv preprint* 2017, **first posted online June 20th 2017** doi: <http://dx.doi.org/10.1101/151811>.

12. Ramsey PH, Ramsey PP: **Optimal Trimming and Outlier Elimination Article in Journal of modern applied statistical methods**. *Journal of Modern Applied Statistical Methods* 2007, **6**:355-360.

13. Wurtz P, Kangas AJ, Soininen P, Lawlor DA, Davey Smith G, Ala-Korpela M: **Quantitative serum NMR metabolomics in large-scale epidemiology: a primer on –omic technology.** . *American Journal Epidemiology* 2017.
